# A predictive patient-specific computational model of coronary artery bypass grafts for potential use by cardiac surgeons to guide selection of graft configurations

**DOI:** 10.3389/fcvm.2022.953109

**Published:** 2022-09-27

**Authors:** Krish Chaudhuri, Alexander Pletzer, Nicolas P. Smith

**Affiliations:** ^1^Auckland Bioengineering Institute, The University of Auckland, Auckland, New Zealand; ^2^Green Lane Cardiothoracic Surgical Unit, Auckland City Hospital, Auckland, New Zealand; ^3^New Zealand eScience Infrastructure, Wellington, New Zealand; ^4^School of Mechanical, Medical and Process Engineering, Queensland University of Technology, Brisbane, QLD, Australia

**Keywords:** coronary artery bypass graft, stenosis, haemodynamics, blood flow, computational model, 1D model, surgical planning, composite graft

## Abstract

Cardiac surgeons face a significant degree of uncertainty when deciding upon coronary artery bypass graft configurations for patients with coronary artery disease. This leads to significant variation in preferred configuration between different surgeons for a particular patient. Additionally, for the majority of cases, there is no consensus regarding the optimal grafting strategy. This situation results in the tendency for individual surgeons to opt for a “one size fits all” approach and use the same grafting configuration for the majority of their patients neglecting the patient-specific nature of the diseased coronary circulation. Quantitative metrics to assess the adequacy of coronary bypass graft flows have recently been advocated for routine intraoperative use by cardiac surgeons. In this work, a novel patient-specific 1D-0D computational model called “COMCAB” is developed to provide the predictive haemodynamic parameters of functional graft performance that can aid surgeons to avoid configurations with grafts that have poor flow and thus poor patency. This model has significant potential for future expanded applications.

## 1 Introduction

Coronary artery disease is the leading cause of mortality and morbidity worldwide (1). Coronary artery bypass grafting (CABG) operations have been performed as a treatment for this disease for over 60 years (2). There has been recent interest in the exclusive use of arterial grafts as conduits for this operation, in particular bilateral internal mammary arteries (BIMA) and radial arteries (RA), on account of their longevity (3, 4). However, there still exists significant variability in grafting strategy among cardiac surgeons and there remains controversy as to the optimal grafting configuration for a given patient (5). This debate is due to the complex biophysics governing the dynamics of a particular individual’s coronary circulation and the resulting degree of uncertainty facing a surgeon. Bypass grafts that have poor flow can occlude early leading to recurrent angina and need for reintervention (6).

Surgeons have attempted to decrease uncertainty using haemodynamic metrics to assess stenoses and grafts. These include functional assessments of coronary stenoses with fractional flow reserve (FFR) or instantaneous wave-free ratio (iFR) and functional assessment of bypass grafts using intraoperative transit-time flowmetry (TTFM) (7).

Transit-time flowmetry (TTFM) utilises an ultrasound flow probe that is placed around a coronary artery bypass graft intraoperatively to verify satisfactory blood flows. The probe contains proximal and distal transducers that enable the calculation of the transit time of reflected ultrasonic signals that are determined by the velocity of blood flow in the bypass graft (8). From the measurement of transit time, the flowmeter can deduce the transient volumetric blood flow. The four main parameters that are displayed in real time which indicate adequacy of graft blood flow are all derived from the measured graft flow waveforms when coupled to an ECG monitor. These are as follows: mean graft flow (MGF), pulsatility index (PI), diastolic filling (DF), and percentage back flow (BF) (9).

Mean graft flow is the mean graft flow, *Q*_*mean*_, in ml/min and for arterial grafts will be determined by the diameter of the graft and native coronary artery, quality of the outflow bed, as well as the anastomosis and presence of any graft spasm (10). A MGF > 15 ml/min is indicative of a satisfactorily performing bypass graft, although flows should be ideally greater than 20 ml/min for left internal mammary artery (LIMA) grafts (11). PI is calculated as the difference in maximum, *Q*_*max*_, and minimum, *Q*_*min*_, flows divided by the MGF, *Q*_*mean*_, such that $P\,I=\frac{Q_{m\,a\,x}-Q_{m\,i\,n}}{Q_{m\,e\,a\,n}}$, and is indicative of flow and resistance through the graft. Ideally, PI should be less than 3, particularly for grafts to the LV territory but a PI less than 5 is acceptable (12). Both MGF and PI have been strongly correlated with graft patency and clinical outcomes (13, 14).

Back flow is the percentage of reverse flow that occurs in a bypass graft and is graphically represented by the area under the horizontal axis on the flow-time curve. Studies have shown that a reverse flow of ≥3.0% is associated with competitive flow and thus indicates that BF can be a predictor of early graft failure (7, 15). Reverse flow will also result in a more negative minimum flow and thus is closely related to PI (11). DF is the percentage of total graft flow that occurs during diastole, or percentage of diastolic coronary filling, such that $D\,F=\frac{Q_{d\,i\,a\,s\,t\,o\,l\,e}}{Q_{s\,y\,s\,t\,o\,l\,e}+Q_{d\,i\,a\,s\,t\,o\,l\,e}}$, and this should be higher for grafts to the left side as the left coronary has a more diastolic dominant pattern with typical values of at least 60% for grafts to the left and 50% for grafts to the right coronary targets (16). However, DF has not been shown to correlate with graft patency outcomes (12).

The site of measurement on the bypass graft can influence the results, and thus, the recommendation is to place the ultrasound probe around the bypass graft at a location that is as close to the distal anastomosis as possible (9). This is due to the fact that the inflow to a graft, if arising from the aorta or an *in situ* internal mammary artery (IMA) will have a systolically dominant pattern, whereas near the anastomosis, it will have a pattern that is more diastolically dominant due to the proximity to the coronary circulation (17). Consequently, the proximal segment of a graft can be influenced by graft capacitive flow that alters the PI and lowers the DF when compared to the distal segment of a graft (18).

Measurement of graft flow intraoperatively is important in total arterial grafting as the use of *in situ*, composite, and sequential grafts based off a limited number of inflow sources often results in the inability to manually test the distal run-off *via* injection of saline into the grafts. Therefore, the routine use of TTFM is recommended for multi-arterial grafting (11). Functional graft failures can be distinguished from technical errors by surgeons such as backwalling an anastomosis based on the TTFM parameters. Although both may have a low MGF < 15 ml/min and high PI > 5, the BF would be close to zero with an anastomotic issue such as thrombosis, anastomotic narrowing, or intimal flap (19). However, with a functional issue, such as competitive flows or steal between limbs of composite grafts or sequential grafts, the BF would be greater than 3% (17). Therefore, if there is an assumption of no technical anastomotic problems and good distal run-off, unsatisfactory MGF and PI are enough to indicate a functional issue.

Although there are a variety of computational models of varying complexity in the literature, to our knowledge, there is no description of a 1D-0D model with the specific purpose of informing a cardiac surgeon of the predictive TTFM parameters that are indicative of graft patency to guide patient-specific selection of grafting configurations. In this work, we demonstrate the haemodynamic predictions arising from a separate and composite total arterial grafting configuration in one patient with severe triple vessel disease.

## 2 Materials and methods

### 2.1 Geometric parameterisation of 1D-0D vessel network topologies

#### 2.1.1 Patient-specific segmentation of coronary artery circulations

**FIGURE 1:**
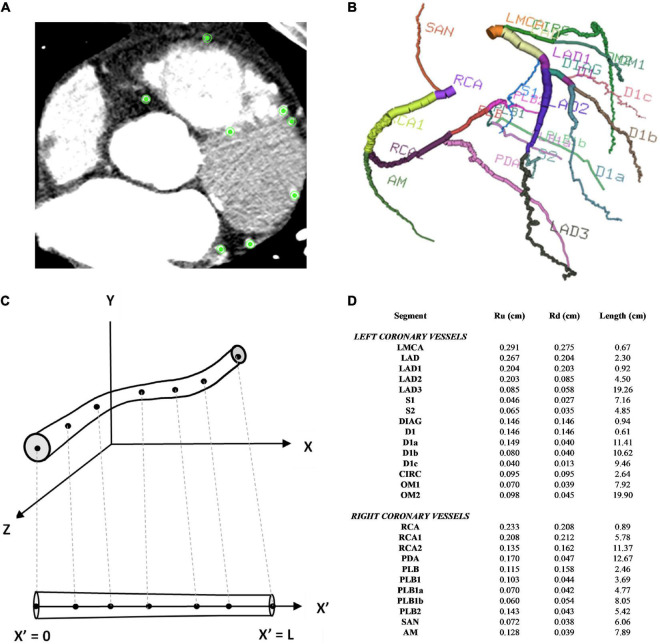
Coronary artery vessel segmentation from CTCA. **(A)** In this example of one axial CTCA slice, there were 8 nodes selected for the centre point of the 8 vessels, as displayed by the green points. This process was repeated for the consecutive slices **(B)** 3D visualisation of 26 coronary vessel segments **(C)** 1D vessel geometry obtained from 3D vessel geometry **(D)** vessel segment dimensions.

Institutional ethics review was obtained to access the CT coronary angiogram study of a patient with multivessel coronary artery disease who had undergone coronary artery bypass grafting. The acquired images were anonymised. The CTCA study had been conducted on a Toshiba 320-slice Aquilion ONE scanner with 640 image slices and an incremental distance between adjacent slices of 0.25 mm. Centreline extraction of the major epicardial and intramyocardial coronary vessels was achieved using a manual method by selecting nodes for the centre points of vessels on consecutive axial image slices using *Horos* (20) (Figure 1A). If the vessel segment was off-axis, then the position of the node was verified against the sagittal and coronal image slices. The rectangular Cartesian coordinates for the nodes were then read into the open-source Visualisation ToolKit (*VTK)* (21) and the centrelines were composed by creating elements to connect the relevant nodes. The linear measurement tool in *Horos* was used to obtain diameter measurements for the vessels at every 4th axial slice to reduce noise associated with multiple measurements within a short axial distance. To construct a disease-free coronary circulation, the sites of stenoses along the native coronary vessels were initially ignored. Their locations were noted and later incorporated into the stenotic circulation model. Similarly, the grafts themselves were ignored and coordinates and dimensions later incorporated for that relevant grafted circulation. Once the diameters of the vessels were obtained, they were converted to radii. A three-dimensional model of the coronary circulation relevant for surgeons was then created using the information from the centreline extraction and vessel radii and these were displayed using *VTK* (Figure 1B). The axial length, *L*, in one dimension for each vessel segment was obtained by mapping each node to the 1D domain by calculating the Euclidean distance between points and the radii measured from the CT scans at the relevant (*X*_*n*_,*Y*_*n*_,*Z*_*n*_) points were similarly mapped onto the 1D vessel segment at each point (X′n) (Figures 1C,D).

#### 2.1.2 Addition of 1D-0D coronary side branches at step-wise vessel tapering

**FIGURE 2:**
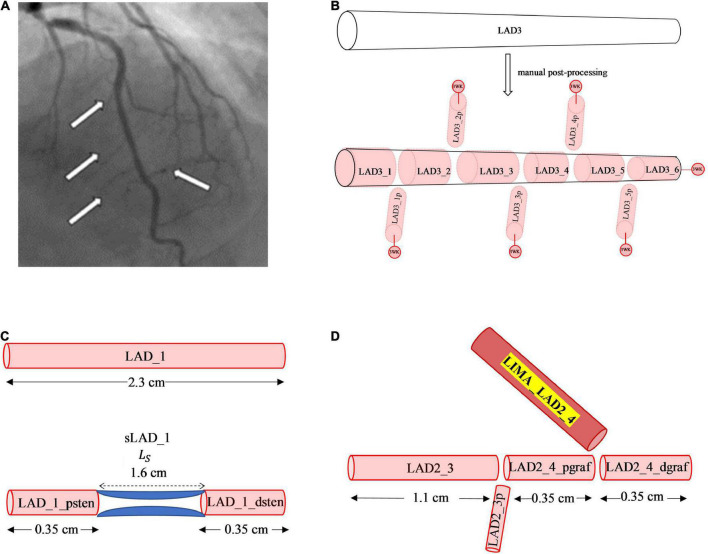
Manual post-processing addition of side branches, terminal 3WK stenosis and grafts. **(A)** Side branches visualised on invasive coronary angiogram. The white arrows on the left-side point to smaller septal perforators off the LAD, whereas the white arrow on the right-side points to a smaller diagonal vessel. **(B)** Stepwise vessel tapering at added coronary side branches: the original segment of the LAD termed the LAD3 that was extracted from the CT coronary angiogram has undergone step-wise tapering with addition of perforating (p) side branches. In this example, the original segment is now made up of 11 new vessel segments with the naming convention for the main vessel and side branches as described. Terminal vessels are connected to lumped parameter 3WK models. **(C)** Example of 1D-0D construction of a stenosis in network topology Adding a 1.6 cm length stenosis, 0.35 cm along the LAD_1 segment, results in 2 new vessel segments termed LAD_1_psten and LAD_1_dsten with new lengths 0.35 cm each. **(D)** Example of 1D representation of end-to-side graft in network topology: Adding a LIMA graft to the LAD3_3 segment, termed the LIMA_LAD2_4, results in the LAD3_3 vessel segment being divided into 2 new segments proximal and distal to the graft site entry which are termed as LAD2_4_pgraf and LAD2_4dgraf, respectively. These 3 vessels now constitute a reverse bifurcation.

Although, the CT coronary angiogram was useful in obtaining the geometry of the major epicardial and intramyocardial coronary artery branches, the resolution was not sufficient to accurately obtain smaller side branches of these vessels. Some of these smaller branches could be visualised from the invasive 2D coronary angiograms (Figure 2A). Accounting for the side branches was considered important for the physiological blood flow and pressure solutions along the major coronary arteries. Using the invasive coronary angiogram as a guide for each patient, truncated 1D branches were added along the original vessels segmented from the CT coronary angiogram. These truncated branches were then connected to a terminal 0D 3-element Windkessel (3WK) lumped parameter model comprising of resistance-capacitance-resistance (RCR) parameters. This lumped model represented the distal microcirculation taking into account the effects of myocardial contraction on the terminal circulation due to the contraction of the ventricles (as described in Section 2.2.6).

Another method that has been used to account for side branches is a physiological 1D leakage model using Murray’s law to taper arterial vessel segments (22). However, an anatomical treatment of the side branches was used in this study as it allowed for a step-wise vessel taper of the main coronary arteries. There exists a normal angiographic tapering of coronary artery vessels (23). Some authors have modelled this continuous taper according to the exponential equations (24, 25). The radius along the vessels obtained from the CT coronary angiogram did not represent a strict tapering due to a localised increase and decrease in radius along the vessel and measurement errors. If a vessel segment has an unfavourable tapering angle, by either having a radius from inlet to outlet that decreases or increases significantly, then the pressure can increase or decrease in a non-physiological manner. To overcome this issue, a step-wise vessel tapering approach was employed for the anatomical models, rather than a continuous taper. The step-wise reduction in vessel diameter was conveniently applied at the added side-branch bifurcations, resulting in a model with each new vessel segment having a constant radius throughout its length (Figure 2B).

This method of step-wise taper at the bifurcations avoided the sudden oscillations and reflections due to geometric non-uniformity seen at step-wise reductions of main vessel diameters in other studies (26, 27). This also resulted in an increase in the number of individual segments in the coronary artery network topology from 26 in the initial coronary artery segmentation to 124 vessel segments post- manual processing.

#### 2.1.3 Addition of generic 1D-0D systemic aortic branches

The addition of coronary artery bypass grafts to the network topology of the native coronary circulation required the consideration of vessels in the systemic circulation, which branch off the sub-branches of the aorta, namely, the left internal mammary artery and the right internal mammary artery. It also required the inclusion of the ascending aorta, itself, as the proximal (inlet) connection of the bypass grafts often attached to this structure. Many of these additional aortic branches and sub-branches could not be segmented from the CTCA due to the limited region of interest in scans involving the thorax. A formal CT angiogram (CTA) that spans the neck and down to the abdomen would be required and this was not available for this particular patient. Therefore, typical radii and lengths of the relevant non-terminal systemic vessels were estimated from the literature, where available (28, 29). The systemic vessels that led to terminal branches were truncated prematurely and coupled to a lumped parameter 3WK RCR model, which unlike the lumped parameter model for the coronary microcirculation did not incorporate the effects of myocardial contraction. The typical dimensions used generically for this patient are reported in Table 1.

**TABLE 1:** Typical dimensions for systemic aortic branches.

Vessel segment name	Abbreviation	Radius (cm)	Length (cm)
Aortic Root I	AO1	1.47	0.5
Aortic Root II	AO2	1.47	0.5
Ascending Aorta	AA	1.47	3
Innominate Artery	INN	0.62	3
Aortic Arch I	ARCH1	1.12	2
Right Common Carotid Artery	RCC	0.4	3
Right Subclavian Artery I	RSCA1	0.48	3
Right Vertebral Artery	RVA	0.2	13.5
Right Subclavian Artery II	RSCA2	0.47	0.75
Right Internal Mammary Artery	RIMA	0.14	18.2
Right Subclavian Artery III	RSCA3	0.45	2.5
Left Common Carotid Artery	LCC	0.37	3
Aortic Arch II	ARCH2	1.07	3.9
Descending Aorta	DA	0.999	5.2
Left Subclavian Artery I	LSCA1	0.423	3
Left Vertebral Artery	LVA	0.2	13.5
Left Subclavian Artery II	LSCA2	0.407	0.75
Left Internal Mammary Artery	LIMA	0.13	18.2
Left Subclavian Artery III	LSCA3	0.38	2.5

Although this approach added 19 more vessel segments to the topology of the coronary circulation model, it was considered preferable for modelling purposes, compared to using generalised transfer functions to represent the LIMA, RIMA, and grafts attached to the ascending aorta. Guala et al. (30) found a 1D-0D lumped parameter model to be more accurate in estimating aortic pressures than generalised transfer functions (30). Therefore, the patient-specific disease-free coronary circulation was nested within a generic systemic aortic circulation. This patient’s network consisted of 1D vessels connected through bifurcations, and the terminal 1D vessels were connected to 0D lumped parameter models. If a patient would have a ramus intermedius artery, then the disease-free coronary circulation would also contain a trifurcation of the left main coronary artery.

#### 2.1.4 Addition of 1D-0D stenoses

The CT coronary angiogram was then used to identify the location of the stenoses and the length of each stenosis. The cardiologist’s interpretation of the invasive coronary angiogram was used to determine the percentage stenosis diameter. These measurements were then applied to the 1D vessel network topology, by renaming the vessel segment proximal to the stenoses as “_psten” and distal to the stenoses as “_dsten” and having the respective lengths of these vessels adjusted. Along the length of the stenosis itself, a 0D lumped parameter model was used to represent the pressure drop across the stenosis (Figure 2C). The mathematical formulations detailing the lumped parameter stenoses are described in Section 2.2.5.3.

#### 2.1.5 Addition of 1D grafting vessel branches

The graft itself represented at least one new vessel segment, and in the case of the *in situ* LIMA or *in situ* RIMA, it was connected to a distal site in the coronary circulation. The existing vessel segment proximal to the site of entry to the graft was renamed “_pgraf,” and the vessel segment distal to the site of entry to the graft was renamed “_dgraf” (Figure 2D). An end-to-side graft would be a reverse bifurcation, a Y-graft would be a normal bifurcation, a sequential graft would be a cross-junction and an I-graft a connector junction.

**FIGURE 3:**
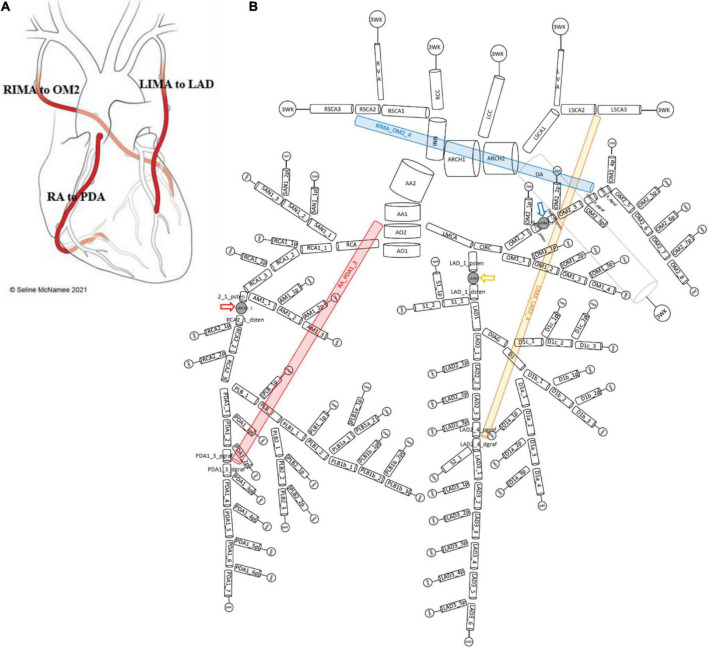
Network topology of grafted circulation separate configuration. **(A)** Separate grafting configuration with *in situ* LIMA to LAD, *in situ* RIMA to OM2 and free RA off aorta to PDA. **(B)** Corresponding vessel network topology with 151 vessel segments and 78 junctions. The RA (red) coming off the aorta required the introduction of a second ascending aorta (AA) vessel segment (AA1 and AA2) and this graft attached to the junction with PDA1_3_pgraf and PDA1_3_dgraf. The LIMA and RIMA vessels were detached from their previous terminal 3WK lumped parameter models in the stenotic circulation network and attached to the coronary circulation. The LIMA (yellow) attached to the junction of LAD2_4_pgraf and LAD2_4_dgraf while the RIMA (blue) attached to the junction of OM2_4_pgraf and OM2_4_dgraf. The three lumped parameter stenoses in the LAD, OM2, and RCA vessels which are shown between LAD_1_psten and LAD_1_dsten (*yellow arrow*), OM2_2_psten and OM2_2_dsten (*blue arrow*), and RCA2_1_psten and RCA2_1_dsten (*red arrow*). The vessel segment data and vessel relations for this topology are in Supplementary File 1.

**FIGURE 4:**
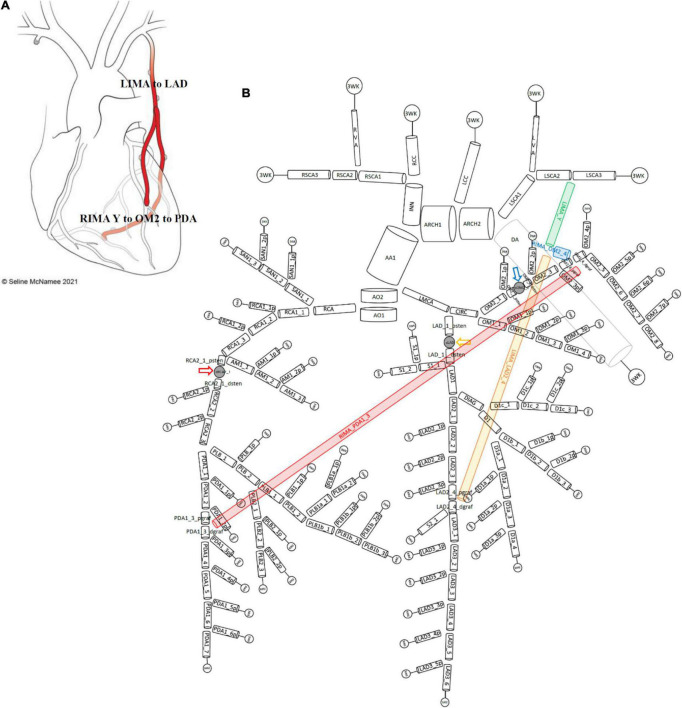
Network topology of grafted circulation composite configuration. **(A)** Composite grafting configuration with *in situ* LIMA to LAD, composite Y RIMA off the LIMA sequentially to OM2 and PDA. **(B)** Corresponding vessel network topology with 151 vessel segments and 78 junctions. As no graft arises from the ascending aorta, there is only one vessel segment (AA1). The common stem of the LIMA (termed LIMA_Y) (green) is the parent inlet to the bifurcation involving the ongoing LIMA (yellow) and the RIMA (blue) that is a Y graft off the LIMA. The ongoing LIMA (LIMA_LAD2_4) terminates at the bifurcation junction of LAD2_4_pgraf and LAD2_4_dgraf while the RIMA (blue) attaches to the junction of OM2_4_pgraf and OM2_4_dgraf and goes on from this cross-junction as the RIMA_PDA1_3 (red) to terminate in a reverse-bifurcation junction with PDA1_3_pgraf and PDA1_3_dgraf. The three lumped parameter stenoses in the LAD, OM2, and RCA vessels which are shown between LAD_1_psten and LAD_1_dsten (*yellow arrow*), OM2_2_psten and OM2_2_dsten (*blue arrow*), and RCA2_1_psten and RCA2_1_dsten (*red arrow*). The vessel segment data and vessel relations for this topology are in Supplementary File 1.

Therefore, a complete 1D network topology of a grafted circulation would contain the followings: a network of branching 1D vessel segments with a single inlet root vessel segment (Aortic Root I) and junctional relationships between vessel segments that could be bifurcations, trifurcations, stenoses, reverse bifurcations, cross-junctions, or connectors. These were coupled to the 0D lumped parameter model of the stenoses, and the terminal vessel segments were coupled to the 0D lumped parameter 3WK model. In total, two different grafting configurations were added into the network topologies containing stenoses to compare a separate (Figures 3A,B) and a composite grafting strategy (Figures 4A,B). The coronary flow through these network topologies could then be predicted using computational fluid dynamics.

### 2.2 Mathematical modelling of 1D-0D vessel networks

#### 2.2.1 Governing 1D Navier–Stokes equations

The 1D Navier–Stokes equations for Newtonian, incompressible fluids were integrated over cross-sectional area to give the following governing equations for a straight elastic artery vessel segment assuming a no-slip boundary condition and axisymmetric flow (31):

Conservation of mass equation:


(1) $$\frac{\partial A}{\partial t}+\frac{\partial Q}{\partial x}=0$$

Conservation of momentum equation:

(2) $$\frac{\partial Q}{\partial t}+\frac{\partial }{\partial x}\,\left(\alpha \,\frac{Q^{2}}{A}\right)+\frac{A}{\rho }\,\frac{\partial P}{\partial x}=-2\,\pi \,\nu \,\frac{\alpha }{\alpha -1}\,\frac{Q}{A}$$

where *A* is the cross-sectional area of the vessel in cm^2^, *Q* is the flow rate in cm^3^/s, *P* is the pressure in dyne/cm^2^, *x* is the axial dimension along the vessel in cm, ρ is the density of blood assumed essentially incompressible with a constant value ρ≈ 1.06 g/cm^3^, ν is the kinematic viscosity ($\nu =\frac{\mu }{\rho }$), μ is dynamic viscosity with μ=0.046 dyn⋅s/cm^2^ (32, 33) and α is the momentum correction coefficient (Coriolis coefficient) that accounts for the non-linear integration of cross-sectional radial velocities and determines the shape of the velocity profile, ϕ(*r*), through Equation (3), *R* is the lumen radius and *r* is the radial coordinate (34):


(3) $$\phi \,\left(r\right)=\frac{\alpha }{2-\alpha }\,\left(1-{\left(\frac{r}{R}\right)}^{\frac{2-\alpha }{\alpha -1}}\right)$$

Some authors summarise the exponential term in Equation (3) using the single parameter, γ, (35):


(4) $$\gamma =\frac{2-\alpha }{\alpha -1}$$

α can take a value between 1 which corresponds to “plug flow” or a flat profile (36) and α=4/3, which is a fully developed parabolic velocity profile that corresponds to Poiseuille’s laminar flow (31). Previous studies regarding velocity profiles have found that in the coronary arteries, the flow is typically flatter corresponding to α=1.1, while α=1 violates the no-slip boundary condition (35). Even in larger arteries such as the aorta, there is a relatively blunt velocity profile (37). For the purposes of this work, the larger systemic aortic branches were assigned a value of α=1.05, while the smaller branches were treated with a similar velocity profile to the coronary arteries α=1.1.

#### 2.2.2 Constitutive Pressure-Area relation

The constitutive equation to close the system of 2 equations with 3 unknowns (*Q*, *P*, *A*) is a relationship between *P* and *A*, based on LaPlace’s law (pressure as a function of area). The transmural pressure, *P*−*P*_*ext*_, which is the difference between internal and external pressure was given by:


(5) $$P-P_{e\,x\,t}=f\,\left(1-\sqrt{\frac{A_{0}}{A}}\right)+P_{0}$$

where:


(6) $$f=\frac{4}{3}\,\frac{E\,h}{R_{0}}=\frac{4}{3}\,\left(k\,1\cdot e^{k\,2\cdot R_{0}}+k\,3\right)$$

where the arterial wall is being modelled as a thin, incompressible, homogenous, isotropic, elastic membrane with elastic modulus *E*, wall thickness *h*, and radius *R* with *R*_0_ and *A*_0_ being the unstressed radius and corresponding area and *P*_0_ the reference pressure. For the 1D vessels that are not terminal vessels affected by an external myocardial contraction or downstream venous pressure, *P*_*ext*_=0. For the terminal 1D vessels that are coupled to a 0D 3WK lumped parameter model, *P*_*ext*_, incorporated downstream venous pressure and for any coronary vessels, the effects of myocardial contraction and downstream venous pressure. The elasticity parameters were represented by *f*, which relied on the empirically derived constants by Stergiopoulos: *k1*, *k2*, and *k3* (33). For the coronary arteries, the values of these constants were *k*1=20.0 (10^6^ g/s^2^/cm), *k*2=−22.5 (cm^–1^), *k*3=86.5 (10^4^/s^2^/cm) and for the larger systemic arteries such as the aorta and its major branches, they were *k*1=3.0 (10^6^ g/s^2^/cm), *k*2=−9.0 (cm^–1^), *k*3=33.7 (10^4^/s^2^/cm) (38).

For the purposes of this work, the systemic arteries with smaller diameters such as the internal mammary arteries were assigned similar values to the coronary arteries as they have similar mechanical properties (39). Radial arteries were also assigned such values, acknowledging that their mechanical properties may be different (40), but their diameters were similar to the other smaller systemic elastic arteries.

#### 2.2.3 1D numerical scheme: The Lax-Wendroff method

The 1D Navier–Stokes equations that have been presented in the (A,Q) system in Equation (1) and Equation (2) can be cast in conservative form as follows:


(7) $$\frac{\partial \text{U}}{\partial t}+\frac{\partial \text{F}}{\partial x}=\text{S}$$

where:


⁢U=[AQ]

F=[Qα⁢Q2A+Aρ⁢P]

S=[0-2⁢π⁢ν⁢αα-1⁢QA]

The 1D finite difference scheme used to solve these partial differential equations the Richtmyer two-step Lax-Wendroff numerical method, which is second-order accurate in both space and time (41, 42).

#### 2.2.4 Inlet pressure boundary condition at root of network

A Dirichlet boundary condition was applied to the inlet of the first vessel at the root of the whole network as each network represented an open loop circuit (43). This boundary condition had pressure values prescribed as the aortic root pressure waveform beyond the aortic valve according to time (Section 2.3.2).

As the implementation of the equations was cast in the (A,Q) system for the Navier–Stokes Equations, the constitutive P-A relation (Equation 5) was rearranged in terms of *A* which allowed inlet pressure *P* to be reformulated in terms of *A*:


(8) $${\text{A}}_{0}^{n+1}=\frac{A_{0}}{{\left(1-\frac{P_{0}^{n+1}-P_{0}}{f}\right)}^{2}}$$

#### 2.2.5 Junctional boundary conditions

The individual arterial vessel segments were connected at junctions by enforcing conservation of mass (flow) and enforcing continuity of total pressure using Bernoulli’s equation to account for kinetic energy losses. Each junction was described in terms of its number of *i* inlet parent vessels denoted *p1* to *pi* and its number of *j* outlet children vessels denoted *c1* to *cj*. Here, the terms “inlet parent” referred to a vessel that carried blood towards the common junction and outlet child referred to a vessel that carried blood away from the junction. Each vessel being considered had a starting mesh gridpoint at *x*_0_ and the end of the vessel at *x_M_*.

Let *Q*_*pi*_*M*__, *P*_*pi*_*M*__, *V*_*pi*_*M*__, *andA*_*pi*_*M*__ denote the flow, pressure, velocity, and area, respectively, of any inlet parent vessel at its end point *x_M_* and *Q*_*cj*_0__, *P*_*cj*_0__, *V*_*cj*_0__, *andA*_*cj*_0__ denote the flow, pressure, velocity, and area, respectively, of any outlet child vessel at its start point *x*_0_, then from the conservation of mass at a junction:


(9) $$\sum Q_{p\,i_{M}}=\sum Q_{c\,j_{0}}$$

From the conservation of total pressure at junction:


(10) $$P_{p\,i_{M}}+\rho \,\frac{1}{2}\,V_{p\,i_{M}}^{2}=P_{c\,j_{0}}+\rho \,\frac{1}{2}\,V_{c\,j_{0}}^{2}$$

Since $V=\frac{Q}{A}$, Equation (10) can be written as:


(11) $$P_{p\,i_{M}}+\rho \,\frac{1}{2}\,{\left(\frac{Q_{p\,i_{M}}}{A_{p\,i_{M}}}\right)}^{2}=P_{c\,j_{0}}+\rho \,\frac{1}{2}\,{\left(\frac{Q_{c\,j_{0}}}{A_{c\,j_{0}}}\right)}^{2}$$

For any junction consisting of *n* vessels, including both parent and child vessels, where *n* = *i* + *j*, one equation will be supplied by the conservation of mass (Equation 9), denoted *F_a_* where *a* = 1, and (*n*−1) equations supplied from the conservation of total pressure, denoted *F_a_* where *a* = 2*ton*, leading to a total system of *n* equations.


(12) $$F_{1}=\sum Q_{p\,i_{M}}-\sum Q_{c\,j_{0}}=0$$

(13) $$F_{a}=P_{p\,i_{M}}+\rho \,\frac{1}{2}\,{\left(\frac{Q_{p\,i_{M}}}{A_{p\,i_{M}}}\right)}^{2}-P_{c_{j\,0}}-\rho \,\frac{1}{2}\,{\left(\frac{Q_{c\,j_{0}}}{A_{c\,j_{0}}}\right)}^{2}=0$$

where *a* = 2,..,*n*

However, at each junction of *n* vessels, there will be 2*n* unknowns as both the flow, *Q*, and areas, *A*, are unknown for each vessel, with the pressure being related to area through the constitutive equation (Equation 5). To reduce the problem at a junction to *n* vessels with *n* unknowns, this requires equations relating the flow and area at the boundaries of the junctions.

**FIGURE 5:**
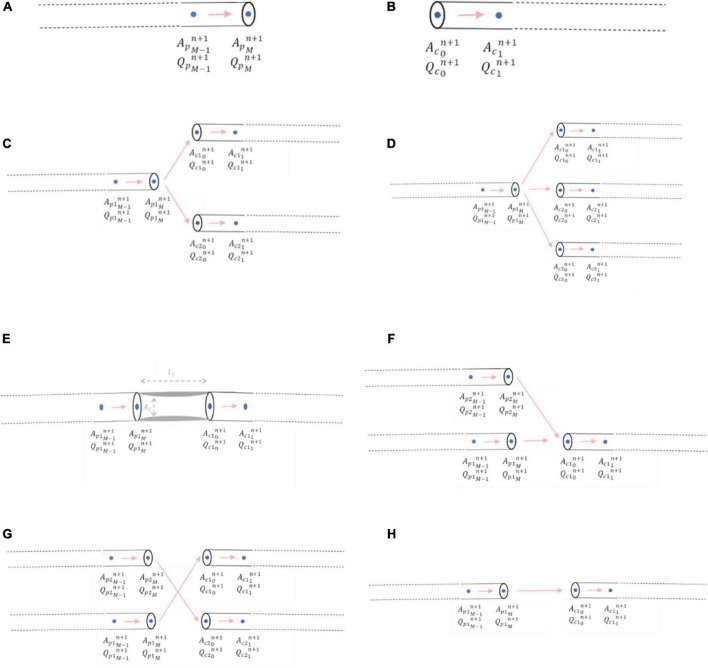
Mathematical modelling of junctional boundary conditions. **(A)** Right-sided junctional boundary condition for parent vessels. **(B)** Left-sided junctional boundary condition for child vessels. **(C)** Bifurcation. **(D)** Trifurcation. **(E)** Stenoses. **(F)** Graft end-to-side (reverse bifurcation). **(G)** Graft sequential (cross-junction): The graft itself entering the junction was *p2*, and the graft exiting the junction was *c2* **(H)** Graft connector.

A right-sided boundary condition applies to the end of an inlet parent vessel, *x_M_* (Figure 5A). Here, a relationship between flows and areas, which involves vessel interior points at the current and previous time steps, was derived using Keller’s Box Method (44):


(14) QpMn+1=-△⁢x△⁢t⁢ApMn+1+△⁢x△⁢t⁢(ApMn+ApM-1n-ApM-1n+1)

-QpMn+QpM-1n+QpM-1n+1

Similarly, a left-sided boundary condition was considered for the start of an outlet child vessel at point *x*_0_ (Figure 5B). The relationship between flows and areas at this left-side boundary condition was:


(15) Qc0n+1=△⁢x△⁢t⁢Ac0n+1-△⁢x△⁢t⁢(Acon+Ac1n-Ac1n+1)

-Qc0n+Qc1n+Qc1n+1

The system of *n* nonlinear equations was solved for each time step for the areas, *A*, of each of the *n* vessels at the junctional boundary by the Newton-Raphson method.

Once the areas were calculated at the boundaries, the pressures and flows could be derived and were executed for each junction type in the network, which are summarised in Table 2.

**TABLE 2:** Summary of junction types that could be encountered in networks.

Junction type	Number of inlet parent vessels (i)	Number of outlet child vessels (j)	Diagram	Disease-Free circulation networks	Stenotic circulation networks	Grafted circulation networks
Bifurcation	1	2		✓	✓	✓
			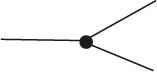			
Trifurcation	1	3		✓	✓	✓
			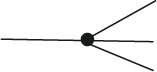			
Stenoses	1	1			✓	✓
			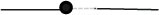			
Graft End-to-Side (Reverse bifurcation)	2	1				✓
			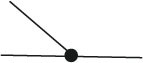			
Graft Sequential (Cross-junction)	2	2				✓
			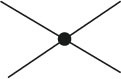			
Graft Connector	1	1				✓
			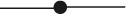			

##### 2.2.5.1 Bifurcations

Bifurcations were the most common junction type encountered in the networks where *i* = 1 and *j* = 2 and consisted of one inlet parent vessel, *p1*, and two outlet children vessels, *c1* and *c2* (Figure 5C). The system of three nonlinear equations that were solved to obtain the areas, pressures, and flows using the Newton-Raphson method was:


(16) Qp⁢1Mn+1-Qc⁢10n+1-Qc⁢20n+1=0

(17) Pp⁢1Mn+1+ρ2⁢(Qp⁢1Mn+1)2(Ap⁢1Mn+1)2-Pc⁢10n+1-ρ2⁢(Qc⁢10n+1)2(Ac⁢10n+1)2=0

(18) Pp⁢1Mn+1+ρ2⁢(Qp⁢1Mn+1)2(Ap⁢1Mn+1)2-Pc⁢20n+1-ρ2⁢(Qc⁢20n+1)2(Ac⁢20n+1)2=0

##### 2.2.5.2 Trifurcations

A trifurcation model was set-up for future use for patients with a left main coronary artery trifurcation dividing into the left anterior descending, the ramus intermedius (intermediate), and circumflex arteries. In Duanmu et al. (32), this was treated as two bifurcations (32); however, in this work, a separate trifurcation model was constructed as it kept the options open to also model a jump-graft that could occur more distally in the coronary circulation, which would not be satisfactorily modelled as two bifurcations.

For a trifurcation, *i* = 1 and *j* = 3 and thus the junction consisted of one inlet parent vessel, *p1*, and three outlet children vessels, *c1*, *c2* and *c3* (Figure 5D).

The system of four nonlinear equations to obtain the areas, pressures, and flows using the Newton-Raphson method was:


(19) Qp⁢1Mn+1-Qc⁢10n+1-Qc⁢20n+1-Qc⁢30n+1=0

(20) Pp⁢1Mn+1+ρ2⁢(Qp⁢1Mn+1)2(Ap⁢1Mn+1)2-Pc⁢10n+1-ρ2⁢(Qc⁢10n+1)2(Ac⁢10n+1)2=0

(21) Pp⁢1Mn+1+ρ2⁢(Qp⁢1Mn+1)2(Ap⁢1Mn+1)2-Pc⁢20n+1-ρ2⁢(Qc⁢20n+1)2(Ac⁢20n+1)2=0

(22) Pp⁢1Mn+1+ρ2⁢(Qp⁢1Mn+1)2(Ap⁢1Mn+1)2-Pc⁢30n+1-ρ2⁢(Qc⁢30n+1)2(Ac⁢30n+1)2=0

##### 2.2.5.3 Stenoses

A stenosis was considered as a percentage diameter narrowing, “%*stenosis*,” in an artery leading to a reduction in cross-sectional area, *A_S_*, over a certain length, *L_S_*. Here, *i* = 1 and *j* = 1 and thus the junction consisted of one inlet parent vessel, *p1*, and one outlet child vessel, *c1* which were not in direct continuity, but separated by a length, *L_S_*, on account of the lumped parameter stenosis (Figure 5E).

However, unique to this junction was the additional lumped parameter pressure drop across the stenosis, △*P*_*stenosis*_. Therefore, the conservation of total pressure upstream and downstream of the stenosis could be written as:


(23) Pp⁢1Mn+1+ρ2⁢(Qp⁢1Mn+1)2(Ap⁢1Mn+1)2=Pc⁢10n+1+ρ2⁢(Qc⁢10n+1)2(Ac⁢10n+1)2+△⁢Ps⁢t⁢e⁢n⁢o⁢s⁢i⁢s

The general form of △*P*_*stenosis*_ was given by:


(24) $$△\,P_{s\,t\,e\,n\,o\,s\,i\,s}=a_{v}\,Q+a_{t}\,Q\,\left|Q\right|+a_{u}\,\frac{\partial Q}{\partial t}$$

and thus △*P*_*stenosis*_ comprised a viscous term with coefficient *a_v_*, a turbulence term with coefficient *a_t_* and an inertial term with coefficient *a_u_*.

In more detailed form:


(25) △⁢Ps⁢t⁢e⁢n⁢o⁢s⁢i⁢s=μ⁢Kv2⁢π⁢R03⁢Q+ρ⁢Kt2⁢A02⁢(A¯0AS-1)2⁢Q⁢|Q|+Ku⁢Lu⁢∂⁡Q∂⁡t

where:

*Q* is the flow rate.

*A*_0_is the unstressed cross-sectional area proximal/distal to the stenosis (*R*_0_is the corresponding radius).

*A*_*S*_is the minimal cross-sectional area of stenosis (*R_S_* is the corresponding radius), that was calculated from the percentage diameter stenosis:


$$\%stenosis=\left(1-\frac{A_{S}}{A_{0}}\right)\times 100$$

(26) $$\frac{A_{0}}{A_{S}}=\frac{100}{100-\%stenosis}$$

*L_S_* is the length of the stenosis.

ρ=1.06 g/cm^3^ is the blood density.

μ=0.046 dyn⋅s/cm^2^ is the dynamic viscosity of blood.

The following constants have been adapted from various models in the literature (45, 46):

$K_{v}=4\,\frac{\alpha }{\alpha -1}\,\frac{L_{a}}{R_{0}}\,{\left(\frac{{\bar{A}}_{0}}{A_{S}}-1\right)}^{2}$ where α is the momentum correction, or Coriolis coefficient set at 1.1 for this work, as Poiseuille flow is not assumed:


$$L_{a}=0.83\,L_{s}+3.28\,R_{s}$$

$$K_{t}=1.52$$

$$K_{u}=1.2$$

$$L_{u}=\frac{\rho }{\pi }\,\int_{0}^{L_{S}}\frac{1}{R\,{\left(x\right)}^{2}}\,dx$$

When grafts were added into the networks containing stenoses, three other potential junctional boundary conditions required consideration: reverse bifurcations, sequential cross-junctions, and simple connector junctions.

##### 2.2.5.4 Reverse bifurcations

An end-to-side graft that connected to a native coronary artery was considered to be part of a reverse bifurcation, where *i* = 2 and *j* = 1, with 2 inlet parent vessels, *p1* and *p2* (one of which was the native coronary artery proximal to the entry point of the graft and the other was the graft itself) and 1 outlet child vessel, *c1*, which was the native coronary artery distal to the entry point of the graft (Figure 5F).

The system of three nonlinear equations that were solved to obtain the areas, pressures, and flows using the Newton-Raphson method was:


(27) Qc⁢10n+1-Qp⁢1Mn+1-Qg0n+1=0

(28) Pp⁢1Mn+1+ρ2⁢(Qp⁢1Mn+1)2(Ap⁢1Mn+1)2-Pc⁢10n+1-ρ2⁢(Qc⁢10n+1)2(Ac⁢10n+1)2=0

(29) Pp⁢2Mn+1+ρ2⁢(Qp⁢2Mn+1)2(Ap⁢2Mn+1)2-Pc⁢10n+1-ρ2⁢(Qc⁢10n+1)2(Ac⁢10n+1)2=0

##### 2.2.5.5 Sequential cross-junctions

A side-to-side graft that connects to one native coronary artery before proceeding to connect to another was considered to be a cross-junction, where *i* = 2 and *j* = 2, with 2 inlet parent vessels, *p1* and *p2* (one of which was the native coronary artery proximal to the entry point of the graft and the other was the graft itself), and 2 outlet children vessels, *c1* and *c2* [one of which was the native coronary artery distal to the entry point of the graft and the other was the ongoing graft as it headed to another coronary artery segment (Figure 5G)]. The conservation of total pressure at a cross-junction behaves like a trifurcation (47) instead of the general form described for the other inlet parent and outlet child vessels in Equation (13). The system of four nonlinear equations that were solved to obtain the areas, pressures, and flows using the Newton-Raphson method was:


(30) Qp⁢1Mn+1+Qp⁢20n+1-Qc⁢10n+1-Qc⁢20n+1=0

(31) Pp⁢1Mn+1+ρ2⁢(Qp⁢1Mn+1)2(Ap⁢1Mn+1)2-Pc⁢10n+1-ρ2⁢(Qc⁢10n+1)2(Ac⁢10n+1)2=0

(32) Pp⁢1Mn+1+ρ2⁢(Qp⁢1Mn+1)2(Ap⁢1Mn+1)2-Pp⁢20n+1-ρ2⁢(Qp⁢20n+1)2(Ap⁢20n+1)2=0

(33) Pp⁢1Mn+1+ρ2⁢(Qp⁢1Mn+1)2(Ap⁢1Mn+1)2-Pc⁢30n+1-ρ2⁢(Qc⁢20n+1)2(Ac⁢20n+1)2=0

##### 2.2.5.6 Connectors

Connector boundary conditions would be required if grafts are lengthened by an end-to-end anastomosis such as in I-graft (48) (Figure 5H). As long as there is no abrupt decrease in radius between the two vessels, oscillations should not occur as previously explained in the issues of step-wise tapering along a vessel (Section 2.1.2). If the radii of the vessels are similar, then the connector boundary condition would not be required and the two vessel segments could be considered as just one vessel segment with increased length and the same radius.

The system of two nonlinear equations to obtain the areas, pressures, and flows using the Newton-Raphson method was:


(34) Qg⁢1Mn+1-Qg⁢20n+1=0

(35) Pg⁢1Mn+1+ρ2⁢(Qg⁢1Mn+1)2(Ag⁢1Mn+1)2-Pg⁢20n+1-ρ2⁢(Qg⁢20n+1)2(Ag⁢20n+1)2=0

#### 2.2.6 Outlet pressure boundary conditions at terminal vessel outlets

**FIGURE 6:**
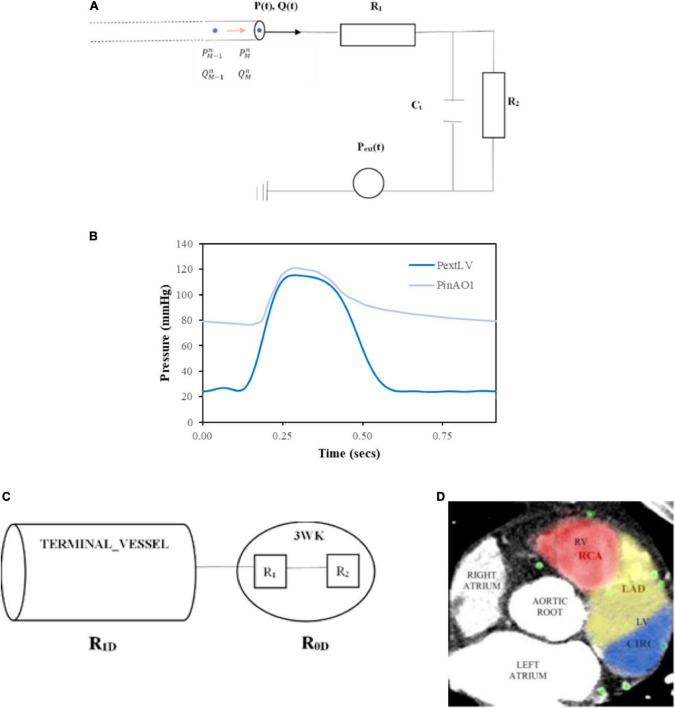
Inlet and terminal outlet parameters. **(A)** The inlet pressure prescribed at the aortic root (PinAO1) is depicted here over 1 cardiac cycle with time period 0.917 s and the external pressure (PextLV) incorporating the effects of LV myocardial contraction and downstream venous pressure is displayed here for one cardiac cycle of time period 0.917 s. **(B)** 3WK RCR electrical analogue where *R*_1_ is the proximal arteriolar resistance, *C_t_* is the arterial compliance through the capacitor and *R*_2_is the distal arteriolar resistance. **(C)** Resistances in terminal outlet vessels. **(D)** Calculating patient-specific distribution of myocardial territory blood flow by examining distribution of coronary vessels in consecutive axial slices from the CT coronary angiogram and the area of distribution was calculated for each myocardial perfusion territory.

The terminal arteries were each connected to a vascular bed of smaller vessels which were represented by a 0D lumped parameter model with resistance and capacitance parameters as an electric circuit analogue for the downstream microvascular tree. This was accomplished with the use of a 3WK RCR (49) (refer to Figure 6A).

As the microvascular network for the coronary arteries is embedded in the myocardium, there is an external pressure, *P*_*ext*_, that is applied to these vessels which is the combination of ventricular extravascular pressure, representing the effects of myocardial contraction on the coronary vessels, and downstream venous capillary pressure. For the systemic arteries such as the aortic sub-branches, *P*_*ext*_ is only comprised of the downstream venous capillary pressure. The computational expense of using a more sophisticated RCRCR model for the coronary circulation as used by other authors (35, 38, 50) was not considered as being beneficial for this work as the focus was on mean flows and pressures in the circulation. Others that have used a 3-element model for the coronary microvasculature have demonstrated the ability to simulate physiological coronary haemodynamics but acknowledged that additional elements could improve the flow signals particularly during early diastole where inertia plays a role (51).

The 3WK RCR equation for the electrical circuit in Figure 6A is as follows:


(36) $$P\,\left(t\right)=P_{e\,x\,t}\,\left(t\right)+\left(R_{2}+R_{1}\right)\,Q\,\left(t\right)+R_{2}\,C_{t}$$

$$\,\left(\frac{d\,P_{e\,x\,t}\,\left(t\right)}{d\,t}-\frac{d\,P\,\left(t\right)}{d\,t}+R_{1}\,\frac{d\,Q\,\left(t\right)}{d\,t}\right)$$

The numerical discretisation of Equation (36):


(37) PMn=Pe⁢x⁢tMn+(R2+R1)⁢QMn+R2⁢Ct

(Pe⁢x⁢tMn+1-Pe⁢x⁢tMn△⁢t-PMn+1-PMn△⁢t+R1⁢QMn+1-QMn△⁢t)

Rearranging in terms of $Q_{M}^{n+1}$:


(38) $$Q_{M}^{n+1}=Q_{M}^{n}+\frac{P_{M}^{n+1}-P_{M}^{n}}{R_{1}}+\frac{△\,t\,P_{M}^{n}}{R_{1}\,R_{2}\,C_{t}}-\frac{△\,t\,\left(R_{2}+R_{1}\right)\,Q_{M}^{n}}{R_{1}\,R_{2}\,C_{t}}$$

-Pe⁢x⁢tMn+1-Pe⁢x⁢tMnR1-△⁢t⁢Pe⁢x⁢tMnR1⁢R2⁢Ct

Incorporating Equation (38), the right-sided boundary condition (Equation 14), and P-A relation (Equation 5), the outlet boundary condition was solved for $P_{M}^{n+1}$ using a fixed point iterative scheme, similar to that described elsewhere (52).

### 2.3 Implementation of computational models

#### 2.3.1 Python programming language

The computational models based on the mathematical formulations of Section 0 were implemented in the *Python* computer programming language (53). A standalone package called “COMCAB” was created containing various modules with solvers for the vessel segments, network boundary conditions, as well as visualisation modules and other output modules.

The *Python* language contains data structures that are well suited for the problem, notably the requirement to support a rich set of vessel topologies, comprising of bifurcations, trifurcations, stenoses, and grafts as detailed in Section 2.2.5. The vessel topology was represented using dictionary-type data structures, which were encapsulated in an object-oriented approach that abstracts many of the implementation details. A simple format for entering the vessel topology in a configuration file was developed, which describes how vessels connect to each other. Objects allow one to query the connectivity of a vessel to its parents and its children.

A potential drawback of choosing Python is that the code can be slow. Initial profiling of the code indicated that most of the execution time was spent in reconciling boundary conditions between connecting vessels. To improve the execution speed, Numba decorators were added to the performance critical code, which automatically translate Python source code to C compiled language. This resulted in a 4.7× speedup over the pure Python code.

The decision to undertake this approach rather than use existing packages was based on the requirement to tailor the coding to the specific investigative models used in this project. Existing open-source software such as the *STARFiSh* flow solver (54) and VaMPy (52) did not contain sufficient detail for junctions that were applicable to grafting strategies while SimVascular (55) did not allow for virtual surgical planning and their assignment of outlet parameters was by “trial and error” and thus considered too manually intensive.

A staged approach was employed for the computational modelling, commencing with the assignment of parameters for the disease-free coronary circulation, then implementing stenoses to represent the diseased coronary circulation and finally incorporating two bypass grafting configurations to obtain grafted models of the circulations.

#### 2.3.2 Inlet pressure at aortic root

A generic inlet pressure was assigned at the root of the whole network, which was the first vessel segment in the aortic root (AO1). The time period of the cardiac cycle for the pressure was *T* = 0.917 s, which corresponds to a resting heart rate of approximately 65 beats per minute. The inlet pressure waveform was obtained from the literature (56) (Figure 6B).

#### 2.3.3 Outlet pressure at terminal outlets

The outlet pressure, *P*_*ext*_, represented the downstream capillary venous pressure for systemic aortic branch outlets and the additional effects of myocardial contraction on the coronary outlet vessels. For the coronary outlets, this pressure transient was in synchronisation with the inlet pressure waveform, and thus, the cardiac cycle time period was also *T* = 0.917 s. Typical Pe⁢x⁢tL⁢V⁢(t) data incorporating left ventricular (LV) myocardial contraction and downstream capillary venous pressure (56) are demonstrated in Figure 6B.

Certain coronary vessels terminate in the right ventricle myocardium or septal myocardium, and the force of contraction is less than the left ventricle (57), resulting in lower external pressures (*P*_*ext*_ values). To capture this effect, the right ventricular external pressure, Pe⁢x⁢tR⁢V, was set at:


(39) Pe⁢x⁢tR⁢V⁢(t)=0.2×Pe⁢x⁢tL⁢V⁢(t)

The septal external pressure,Pe⁢x⁢tS⁢E⁢P⁢T⁢A⁢L modelled the influence of the left ventricle and right ventricle on arteries in the septal region and was set at:


(40) Pe⁢x⁢tS⁢E⁢P⁢T⁢A⁢L⁢(t)=0.6×Pe⁢x⁢tL⁢V⁢(t)

For the systemic arterial microvascular tree, no such myocardial squeeze was applied, and thus, *P*_*ext*_ solely comprised of the venous capillary pressure, which was set at 1 mmHg (58).

#### 2.3.4 Assigning resistance-capacitance-resistance values at terminal outlets

Assigning appropriate values at individual terminal outlets for the proximal resistance, *R*_*1*_, distal resistance, *R*_*2*_, and capacitance,*C_t_* (Section 2.2.6) was important as these parameters dictate the pressures and flows throughout the upstream vessel network branches.

The smallest diameter of the terminal vessels originally segmented from the CT coronary angiogram (refer to Section 2.1.1) (Figure 1D) was 260 μ*m*. Typically, the largest resistance is contributed by arterioles 150 to 300 μ*m* in diameter, whereas the smaller distal arterioles and distal capillaries contribute less resistance (59). Therefore, for the purposes of this work, given the detail achieved by the geometric segmentation of terminal vessels, the proximal arteriolar resistance, *R*_*1*_, would often be larger than the distal arteriolar resistance, *R*_*2*_ in the 3WK model for coronary vessels. However, for the systemic aortic branches whose truncated diameters were in range of 0.26 to 2 cm (i.e., 2,600 to 20,000 μ*m*) (Table 1), the proximal resistance, *R*_*1*_, would represent an arterial resistance that would tend to be less than the distal arteriolar resistance, *R*_*2*_.

##### 2.3.4.1 Total lumped resistances at the terminal outlets

Each terminal vessel leading to a 3WK has a 1D resistance, *R*_*1D*_, arising from the length and radius of that vessel segment, and this was coupled to the total lumped parameter 0D resistance, *R*_*0D*_, such that the total resistance arising from the terminal vessel segment, *R*_*terminal*_, is given by:


(41) $$R_{t\,e\,r\,m\,i\,n\,a\,l}=R_{1\,D}+R_{0\,D}$$

Since the two resistances in the lumped parameter 3WK RCR are also in series, the total lumped parameter resistance is: *R*_0*D*_=*R*_1_ + *R*_2_ (Figure 6C).

Since, $\text{Q}=\frac{△\,\text{P}}{R}$, the flow in the terminal vessel segment will be given by the difference in pressure from the start of the vessel segment to the external pressure prescribed at the end, *P*_*ext*_.

Considering mean flows, ${\bar{Q}}_{t\,e\,r\,m\,i\,n\,a\,l}$:


$${\bar{Q}}_{t\,e\,r\,m\,i\,n\,a\,l}=\frac{{\bar{P}}_{t\,e\,r\,m\,i\,n\,a\,l_{s\,t\,a\,r\,t}}-{\bar{P}}_{e\,x\,t}}{R_{1\,D}+R_{0\,D}}$$

$$R_{0\,D}=\frac{{\bar{P}}_{t\,e\,r\,m\,i\,n\,a\,l_{s\,t\,a\,r\,t}}-{\bar{P}}_{e\,x\,t}}{{\bar{Q}}_{t\,e\,r\,m\,i\,n\,a\,l}}-R_{1\,D}$$

(42) $$R_{1}+R_{2}=\frac{{\bar{P}}_{t\,e\,r\,m\,i\,n\,a\,l_{s\,t\,a\,r\,t}}-{\bar{P}}_{e\,x\,t}}{{\bar{Q}}_{t\,e\,r\,m\,i\,n\,a\,l}}-R_{1\,D}$$

This provides an expression to calculate the individual lumped parameter resistances, *R*_*1*_ and *R*_*2*_ in terms of 1D features of the terminal vessel segments, including the mean pressure at the start of the vessel, the mean external pressure applied at the outlet (as described in Section 2.3.3), the 1D resistance of the vessel segment itself, and the mean flows.

##### 2.3.4.2 Proximal resistance at the terminal outlets

The proximal lumped parameter resistance, *R*_1_, in the 3WK RCR model was set as the characteristic impedance, *Z*_0_, of the terminal vessel to minimise wave reflections while taking into account the terminal vessel diameter (radius) (60, 61):


(43) $$R_{1}=\frac{\rho \,c_{0}}{A_{0}}$$

where *c*_0_ is the wave-speed and is defined by the Moens-Korteweg Equation, based on the arterial wall elasticity parameters defined in Equation (5) and Section 2.2.2:


(44) $$c_{0}^{2}=\frac{f}{2\,\rho }=\frac{2}{3\,\rho }\,\left(k_{1}\cdot e^{k_{2}\cdot r_{0}}+k_{3}\right)$$

For the systemic aortic branch outlets, the calculation of *R*_1_ would be identical between different patients when using a common geometric parameterisation. However, for the coronary outlets, the calculation of *R*_1_ would be patient-specific, based on the segmented vessel radii.

##### 2.3.4.3 Distal resistance at the terminal outlets

The method implemented for setting the distal lumped parameter resistance, *R*_2_, required considering a rearrangement of Equation (42):


(45) $$R_{2}=\frac{{\bar{P}}_{t\,e\,r\,m\,i\,n\,a\,l_{s\,t\,a\,r\,t}}-{\bar{P}}_{e\,x\,t}}{{\bar{Q}}_{t\,e\,r\,m\,i\,n\,a\,l}}-R_{1\,D}-R_{1}$$

From above, the external pressure was set according to the specific systemic or coronary outlet (Section 2.3.3), and the *R*_1_ was calculated from Equation (43). Furthermore, the 1D resistance of a vessel was calculated using a similar equation described in the literature (60) by applying the relationship defined in Equation (4):


(46) $$R_{1\,D}=2\,\frac{\alpha }{\alpha -1}\,\mu \cdot \frac{L}{\pi \,R^{4}}$$

Estimating the mean pressure at the start of the terminal vessel required consideration of the routes of blood flow to the terminal vessel from the root vessel in the network, an approximation of the areas of the upstream vessels using the unstressed areas as well as the mean flows in the upstream vessel, as described in the literature (62).

Due to the loss of information from the premature truncation of systemic aortic branches, the vessel geometry could not be relied upon to dictate the distal resistance values, and thus, the eventual calculated flows. Therefore, to maintain accuracy, an estimate of mean flows in the vessels was obtained by prescribing a percentage of total flow in the network for each terminal vessel, based on typical values found in the literature, at rest such as 19.3% of the total cardiac output going to the innominate artery, 5.2% to the left common carotid artery, 6.4% to the left subclavian artery, and approximately 65% to the descending thoracic artery (63, 64). This was distributed as 11.3% to the right common carotid artery (RCC), 1.0% to the right vertebral artery (RVA), 2.4% to the right internal mammary artery (RIMA), 4.6% to the right subclavian artery III (RCSA 3) (where innominate artery = RCC + RVA + RIMA + RSCA3 = 19.3%), 1.0% to the left vertebral artery, 2.2% to the left internal mammary artery, 3.2% to the left subclavian artery III (where left subclavian artery = LVA + LIMA + LSCA3 = 6.4%), and 64.6% to the descending aorta and the remaining 4.5% of the cardiac output to the coronary circulation.

The calculation of the distal resistance for the coronary outlets was more patient-specific and was dictated by the distribution and geometry of the vessels segmented from the CT coronary angiogram of the individual patient. For the individual coronary outlets, the mean flows were estimated as the percentage flow according to the areas of the outlets based on the percentage of myocardial territory perfusion. The total myocardial perfusion of the heart can be divided into the three main regions termed the LAD_*territory*_, CIRC_*territory*_, and RCA_*territory*_ corresponding to the regions subtended by the coronary artery branches of the LAD, CIRC, and RCA, respectively. The percentage of total myocardial blood flow to each territory can also be estimated by examining the distribution of vessels from the CTCA across multiple slices with 4.5% assigned to the myocardium (65) (Figure 6D).

Each terminal coronary outlet was assigned to one of the three main territories. The flows in each outlet were determined by the terminal areas of those outlets, with those outlets with larger areas having more flow through them as they have more area supplied by their distal branching microcirculation outlets, according to an adaptation of Murray’s Law (55):


(47) $${\bar{Q}}_{t\,e\,r\,m\,i\,n\,a\,l}=\frac{{\left(\sqrt{A_{0_{t\,e\,r\,m\,i\,n\,a\,l}}}\right)}^{2.6}}{\sum {\left(\sqrt{A_{0_{a\,l\,l\,\_\,t\,e\,r\,m\,i\,n\,a\,l\,s}}}\right)}^{2.6}}\times {\bar{Q}}_{t\,o\,t\,a\,l\,\_\,t\,e\,r\,r\,i\,t\,o\,r\,y}$$

Therefore, for patient-specific calculations of *R*_*2*_ for the coronary outlets, a cardiac output was applied as well as the percentage of cardiac output represented by myocardial blood flow and the percentages of this myocardial perfusion subtended by each of the coronary territories as estimated from the CTCA. A similar method that allowed for the patient-specific geometry to dictate the assignment of peripheral coronary resistances according to ventricular muscular volume perfusion for each major coronary branch has been described by Morimoto et al. (66).

##### 2.3.4.4 Capacitance at the terminal outlets

The lumped parameter capacitance was estimated by calculating the time constant from the diastolic decay curve of the inlet pressure waveform, which was of the form:


(48) $$P-P_{o\,u\,t}=\left(P_{0}-P_{o\,u\,t}\right)\,e^{\frac{-\left(t-t_{0}\right)}{R_{t\,o\,t\,a\,l}\,C_{t\,o\,t\,a\,l}}}$$

where the time constant is τ=*R*_*total*_*C*_*total*_ and *R*_*total*_ is the total peripheral resistance, *C*_*total*_ is the total arterial compliance, *P*_0_ is the reference pressure in diastole, *t*_0_ is the corresponding time to this reference pressure, and *P*_*out*_ is the outflow pressure (60, 67).

*C*_*total*_ of the network is the sum of the individual terminal outlet capacitances. Therefore, the capacitance of an individual outlet, *C*_*t*_, is related to the lumped resistance at that outlet by:


(49) $$C_{t}=\frac{\tau }{R_{0\,D}}$$

Fitting a curve to the inlet pressure waveform used in this work (Figure 6B), with *p*_0_ = 86.92 mmHg set as the pressure at the start of the diastolic decay curve revealed the time constant to be τ=1.25,since $\frac{1}{\tau }$ = 0.8. This calculated time constant agreed with mean physiological values obtained in human studies where τ=1.33 ± 0.34, range: 0.77 to 1.97 (68).

Therefore, the capacitance (*C_t_*) of each patient-specific terminal lumped parameter model outlet was calculated as:


(50) $$C_{t}=\frac{1.25}{R_{1}+R_{2}}$$

#### 2.3.5 Simulation

##### 2.3.5.1 Vessel segment parameterisation and network relations

From the descriptions thus far, each individual vessel segment in the entire 1D-0D lumped parameter network for a particular patient had parameters assigned in a network (.csv) file and the vessel segment relations described in a network configuration (.cfg) file (Supplementary File 1).

##### 2.3.5.2 Initial conditions

To run the physiological blood flow and pressure solutions over the mesh grid for each vessel segment in the network required supplying initial conditions for ${\text{U}}_{i}^{n}$. Initial flows were set at ${\text{Q}}_{i}^{0}=0$ at every mesh gridpoint *x_i_* from *x*_0_ to *x*_*M*_ for each vessel segment. Initial areas were set at the reference area, *A*_0_ such that ${\text{A}}_{i}^{0}=A_{0}$ at every mesh gridpoint *x_i_* from *x*_0_ to *x_M_* for each vessel segment. To correlate with this, the initial pressures were set at the corresponding reference pressure, *P*_0_ (refer to Section 2.2.2). The value of *P*_0_ was set to the diastolic pressure of the inlet pressure waveform given at the inlet boundary condition at the root of the network. Thus, *P*_0_=76.41 mmHg was set at every mesh gridpoint at time *n* = 0 for each vessel segment.

##### 2.3.5.3 Stability of numerical scheme (Courant–Friedrichs–Lewy condition)

If *L*_*min*_ was the length of the shortest vessel segment in the network of vessels under the consideration for a particular patient, the parameters of the meshgrid for that network were set such that $△\,x<\frac{L_{m\,i\,n}}{2}$to ensure that there were at least 3 gridpoints in each vessel segment. Once the grid-space was set for an individual network simulation, the time step, △*t*, for the numerical scheme was chosen to ensure numerical stability by satisfying the Courant–Friedrichs–Lewy (CFL) condition (52):


(51) $$\frac{△\,t}{△\,x}\leq \frac{1}{\left|V\pm c_{0}\right|}$$

where *V* is the velocity and *c*_0_ is the wave-speed given by Equation (44).

The values that were set for this patient’s networks were: △*x* = 0.1 and, based on this, △*t* = 2.95×10^−5^was chosen to be the largest value possible without violating Equation (51) to ensure both computational efficiency and stability.

##### 2.3.5.4 Execution

To initiate the execution of a particular network model, the vessel segment dimensions and topology were read from the relevant .csv and .cfg files. The inlet pressure at the root of the network was applied to the existing initial conditions, and the solvers run by applying the other boundary conditions to each vessel segment in the network. The numerical solution was calculated over a run of 4 cardiac cycles as the solution was found to converge to dynamic steady state after 3 cardiac cycles. Solutions from the 4th cardiac cycle were stored for each vessel segment at every gridpoint and at 100 equally spaced time intervals in that cardiac cycle in 3D arrays for areas, pressures, and flows. There were 143 vessel segments in the theoretical non-diseased circulation network, 146 vessel segments in the stenotic circulation network, 151 vessel segments in the separate grafting configuration network, and 151 vessel segments in the composite grafting configuration network. The execution time for these network simulations was 55, 62, 74, and 75 min, respectively, on Xeon Broadwell CPUs (2.1GHz), and hence, all 4 networks could be solved using high performance computing within 75 min with embarrassing parallelisation (69).

### 2.4 Output analysis of computational models

#### 2.4.1 Visualisation

Pressure and flows were plotted against distance along vessel segments according to time, *Q*(*x*,*t*) and *P*(*x*,*t*). *VTK* was used for 3D visualisation of the calculated 1D pressures and flows that were interpolated back onto the original 3D geometries of vessels that had been segmented from the CTCA.

#### 2.4.2 Model verification and validation

The computational model was verified and validated by checking the return of realistic pressure and flow waveforms for vessel segments throughout the network tree. This included the resultant flow waveform at the root vessel of the network and the flows down the major systemic aortic branches. The flow and pressure waveforms were verified for the left (LAD and CIRC) and right (RCA and PDA) coronary arteries by comparing with other models and data in the literature. The grafting model was verified by checking the realistic return of the flow and pressure along a LIMA to LAD graft. The models were calibrated to measurements, and a sensitivity analysis was undertaken to determine how relevant parameters in the model influenced the model output (70). This included artificially varying the severity of stenoses in the model to ensure that realistic decreases in pressure were being simulated. Graft performance indices such as MGF, PI, BF, and DF were also verified by checking the return of realistic values. The validation of the grafting models was performed by comparing the predicted MGF and PI of grafts with measurements available in the literature. For validation of the regional myocardial territory perfusion predicted by the non-diseased computational models, the myocardial blood flow per gram of muscle in every coronary territory was calculated to ensure that this measure was greater than 1.5 ml/g/min when no stenoses are present (71). Validation of the stenosis models was achieved by comparing pressure drops across stenoses and their predicted functional significance to existing data in the literature, where it has been noted that at rest, stenoses greater than 85% lead to appreciable decreases in vessel flow (72, 73).

#### 2.4.3 Regional myocardial territory perfusion

Mean flows across the cardiac cycle, ${\bar{Q}}_{i}$, were calculated for each vessel segment at a specified distance, *x*, along the length of the vessel where *x* = *i*△*x*, and *i* is the mesh gridpoint number, by averaging the instantaneous flows, $Q_{i}^{n}$, recorded at 100 equally spaced time intervals, *N*, over the one cardiac cycle:


(52) $${\bar{Q}}_{i}=\frac{\sum_{0}^{N}Q_{i}^{n}}{N}$$

For the calculation of regional myocardial territory perfusion, the mean flows at the end of the vessel at the terminal outlets assigned to a particular territory were summed:


(53) Q¯t⁢e⁢r⁢r⁢i⁢t⁢o⁢r⁢yM=∑Q¯s⁢e⁢g⁢m⁢e⁢n⁢tM

#### 2.4.4 Significance of stenoses

Instantaneous wave-free ratio was chosen to determine the significance of stenoses, as this metric was calculated at rest, in keeping with the simulation. FFR could also have been calculated directly but this would have required a crude estimation of the coronary flow reserve (CFR), which others have assigned CFR as 2.6 (74), but this may differ between specific patients as the estimate of 2.6 applies to non-ischemic hearts (75). Furthermore, as the graft performance parameters that surgeons are familiar with are also calculated at rest, to maintain consistency and avoid the need to run more simulations at hyperaemia, the decision was made to use iFR. This required the consideration of the pressures recorded in the vessel segment proximal and vessel segment distal to the stenosis during the diastolic wave-free period. The mean pressure at the end of the vessel segment proximal to the stenosis was calculated over the diastolic wave-free period as the last 75% in time duration of diastole, ${\bar{P}}_{p\,s\,t\,e\,n_{M}}\,\left(D\,W\,F\right)$. Similarly, the mean pressure at the start of the vessel segment distal to the stenosis was calculated over the diastolic wave-free period,${\bar{P}}_{d\,s\,t\,e\,n_{0}}\,\left(D\,W\,F\right)$. Therefore, the iFR of the particular stenosis, *iFR*_*sten*_, was calculated as follows:


(54) $$i\,F\,R_{s\,t\,e\,n}=\frac{{\bar{P}}_{d\,s\,t\,e\,n_{0}}\,\left(D\,W\,F\right)}{{\bar{P}}_{p\,s\,t\,e\,n_{M}}\,\left(D\,W\,F\right)}$$

#### 2.4.5 Graft performance

The measures of graft performance that most surgeons are familiar with are the mean graft flow, the pulsatility index, the backwards flow, and the diastolic filling percentage as measured on intraoperative TTFM.

The mean graft flow at a specific site, ${\bar{Q}}_{i}$, was calculated using the method used to calculate the mean flows in a vessel segment (Equation 52). It was reported at the end of the graft towards the anastomosis as previous clinical studies had demonstrated the influence of the graft capacitive flow on altering the pulsatility index and lowering the diastolic filling percentage when measurements were taken near the proximal end of the graft (18).

The pulsatility index was calculated at the same site, *i*, by taking into consideration the maximum flow, *Q*_*max_i_*_, minimum flow, *Q*_*min_i_*_, and mean flow, ${\bar{Q}}_{i}$, across the cardiac cycle using the formula:


(55) $$P\,I=\frac{Q_{m\,a\,x_{i}}-Q_{m\,i\,n_{i}}}{{\bar{Q}}_{i}}$$

The diastolic filling percentage was calculated as the blood flow in diastole divided by the total blood flow, whereas the backwards flow fraction was calculated as the percentage of reverse (negative) blood flow during one cardiac cycle as the negative area under the curve (9).

## 3 Results

### 3.1 Verification and validation of vessel segment solutions

**FIGURE 7:**
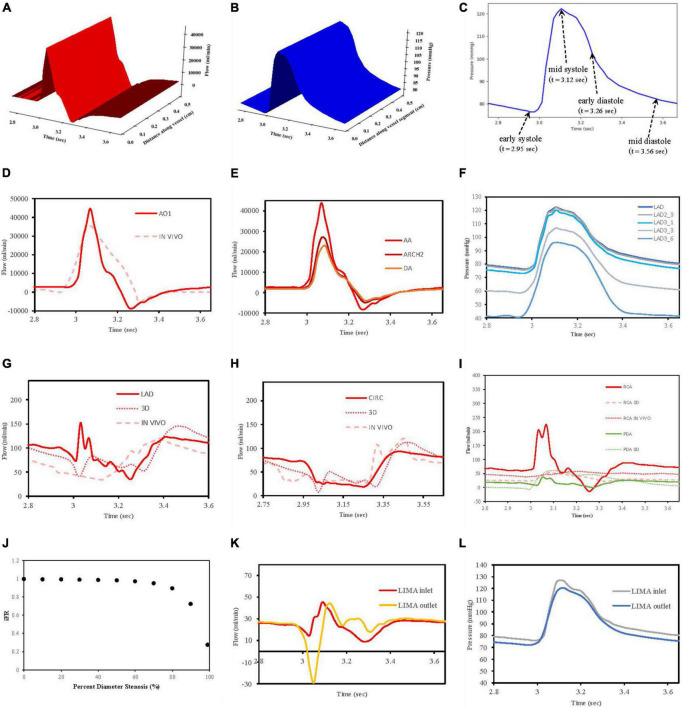
Verification of flow and pressure waveforms in vessels, stenoses and grafts. **(A)** Vessel segment flow according to distance and time. **(B)** Vessel segment pressure according to distance and time. **(C)** Reference times for aortic root pressure. **(D)** Aortic root flow (AO1) compared to *in vivo* data from Bertelsen et al. (76). **(E)** Flows along aorta. **(F)** Pressures along LAD. **(G)** LAD flows compared to 3D model of Kim et al. (50) and *in vivo* data from Duncker and Merkus (80). **(H)** CIRC flows compared to 3D model of Kim et al. (50) and *in vivo* data from Mynard et al. (57). **(I)** RCA and PDA flows compared to 3D RCA flows from Kim et al. (50), *in vivo* RCA flows from Duncker and Merkus (80) and 0D PDA flows from Duanmu et al. (84). **(J)** Pressure drop (iFR) with increasing stenoses diameter. **(K)** LIMA graft inlet and outlet flows. **(L)** LIMA graft pressures at inlet and outlet.

Flows *Q*(*x*,*t*) and pressures *P*(*x*,*t*) were able to be visualised for any of the vessel segments solved in the network according to distance and time to verify that the models were behaving as expected (Figures 7A,B). In total, four reference time points along the inlet aortic root pressure waveform are indicated for early systole, mid systole, early diastole, and mid diastole (Figure 7C).

#### 3.1.1 Flows in the aortic root and major aortic branches

In this work, an inlet pressure was prescribed at the root vessel segment and solved for flow which was different to the strategy employed in VaMpy (52) where an inlet flow was prescribed resulting in pressure calculations. VaMpy used an 18 × 18 Jacobian matrix in their bifurcation solver compared with the 3 × 3 matrix used here. Nevertheless, the inlet flow profile obtained was similar to that obtained from *in vivo* patient data in a study examining flow measurements at the aortic root with cardiac MRI (76) (Figure 7D).

Solving the whole network of vessel segments resulted in realistic flow waveforms along the ascending aorta and the descending thoracic aorta, as described by other computational models in the literature (77, 78). Specifically, with increasing distance away from the aortic root, the flow waveforms in the thoracic aorta demonstrated a decrease in amplitude and a phasic delay shift (Figure 7E). The quantified mean flow at the start of this aortic root segment (equivalent to the cardiac output) was calculated 5.07 L/min as per the prescription of flow at 5 L/min.

#### 3.1.2 Pressures along left anterior descending artery

The decrease in pressures obtained along the length of the left anterior descending artery in a non-diseased circulation (Figure 7F) agreed with the observations reported in a computational model of pulsatile blood flow in the entire coronary tree that was validated by porcine heart models (79).

#### 3.1.3 Flow waveforms in the LAD and CIRC

The obtained flows in the left coronary vessels demonstrated a strong diastolic-dominant waveform with less flow in systole due to the downstream effects of left ventricular myocardial contraction. A direct comparison with the predicted flow waveforms from 3D computational modelling by Kim, Vignon-Clementel et al. (50), for the LAD and CIRC vessels as well as *in vivo* data reported for the LAD (80) and CIRC (57) is shown in Figures 7G,H. The left main coronary artery mean blood flow in the non-diseased circulation was 157.6 ml/min.

#### 3.1.4 Flow waveforms in the RCA and PDA

The right coronary artery had a flow waveform that was significantly less diastolic dominant than the left main coronary artery (81–83). The obtained flows at the right coronary artery inlet overestimated the peak systolic values compared to 3D modelling (50) and *in vivo* data (80) due to neglecting the effects of head loss from the origin off the aorta as detailed by Duanmu et al. (84). However, in the PDA, this effect had diminished which was more important, given that the calculation of the graft performance indices was done near the anastomosis of a graft to the PDA (Figure 7I). The right coronary artery mean blood flow in the non-diseased circulation was 71.40 ml/min.

#### 3.1.5 Regional territory and total myocardial perfusion

The regional myocardial perfusion in each of the three myocardial territories was divided by the calculated mass of myocardium to obtain values of LAD at 1.95; CIRC 1.58, and RCA 1.54 ml/min/g. These values were consistent with *in vivo* data from the literature for coronary circulations without any obstructive disease as being greater than 1.5 ml/g/min (71). The total myocardial blood flow was calculated as 229.04 ml/min, representing 4.52% of the cardiac output as per the prescription of 4.5%.

#### 3.1.6 Stenoses

A relationship between iFR and percent diameter stenosis was explored by increasing the stenosis severity between 0 and 99%, and this verified that the stenosis model was behaving appropriately. A percent diameter stenosis greater than 85% was required to decrease flows significantly in keeping with an *in vivo* clinical study measuring basal myocardial blood flow in 35 patients with single vessel coronary artery disease (73) (Figure 7J).

The stenoses in the LAD, OM2, and RCA led to iFR calculations of 0.93, 0.29, and 0.73. This correlates with the observation in the literature that not all lesions that are 71 to 90% diameter stenosis are functionally significant (85). The stenoses resulted in a decrease in regional myocardial perfusion and total myocardial perfusion when compared to the theoretical non-diseased circulation (Table 3).

**TABLE 3:** Haemodynamic predictions.

Functional Significance of Stenoses							
**Stenosis location**	**Stenosis length (cm)**	**% Diameter stenosis**	**iFR**	**FFR**	**Region**	**Regional perfusion (ml/min)**	**No disease (ml/min)**

LAD	1.6	90	0.93	0.81	LAD	87.48	95.95
OM2	1.2	99	0.29	0.38	CIRC	33.27	61.69
RCA	1.5	90	0.73	0.68	RCA	57.83	71.40
						*178.59*	*229.04*

**Functional Graft Performance**							

**Configuration**	**Region**	**Graft**	**MGF (ml/min)**	**PI**	**BF (%)**	**DF (%)**	**Regional perfusion (ml/min)**

	LAD	LIMA to LAD	24.47	2.98	3.76	70.19	90.80
**Separate**	CIRC	RIMA to OM2	42.79	1.68	0.30	83.37	75.95
	RCA	Radial to PDA	36.60	2.03	0.23	75.77	71.88
							*238.63*
	common	LIMA Y common stem	63.86	1.17	0.00	71.39	−
**Composite**	LAD	LIMA to LAD	6.31	11.33	20.66	56.04	88.40
	CIRC	RIMA to OM2	57.54	1.21	0.00	76.37	74.82
	RCA	RIMA to PDA	15.87	2.59	0.34	64.5	64.04
							*227.26*

#### 3.1.7 Flows and pressures along grafts

The flow and pressure waveforms obtained in the LIMA graft to the LAD at its proximal and distal end (at the anastomosis to the LAD) (Figures 7K,L) were compared to the observations of others. The flow waveform at the inlet of the LIMA demonstrated a systolically dominant pattern as it arises from the left subclavian artery, whereas at the outlet of the LIMA, there was a more diastolically dominant pattern due to the proximity to the coronary circulation at its anastomosis (17). The predicted MGF of the separate LIMA to LAD graft of 24.47 ml/min and PI of 2.98 were within the expected values when compared to a large *in vivo* clinical study measuring TTFM parameters in 333 LIMA to LAD grafts in which MGF was 29.71 ± 20.94 ml/min and PI 2.65 ± 1.01 (86). The predicted MGF for an *in situ* RIMA to OM2 was 42.79 ml/min with PI 1.68, compared to an *in vivo* clinical study by Han et al. (87) with 31 patients in which MGF was 33.4 ± 24.1 ml/min with PI 2.3 ± 0.6 (87). The RA to PDA had MGF of 36.60 ml/min and PI 2.03 which compared well to a clinical study by Onorati et al. (88) involving 69 patients where MGF was 35.9 ± 10.9 ml/min and PI 2.3 ± 1.0 (88). TTFM measurements are taken *in vivo* at a variable site along each graft between different patients and unlike the calculations possible with the computational models cannot be measured at the true origin of the graft or true insertion point at the anastomosis.

### 3.2 Flows and pressures throughout coronary circulation networks

**FIGURE 8:**
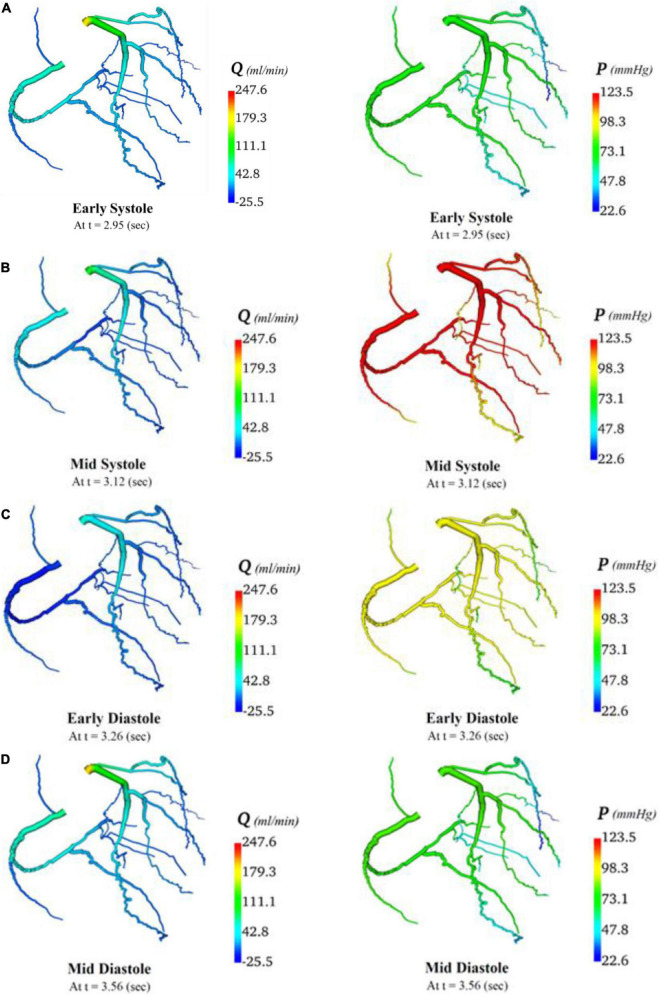
Flows and pressures in theoretical non-diseased coronary artery circulation. The flows (Q) in ml/min and pressures (P) in mmHg are displayed for four time points in the cardiac cycle: **(A)** early systole, **(B)** mid systole, **(C)** early diastole, and **(D)** mid diastole as indicated in Figure 7C.

**FIGURE 9:**
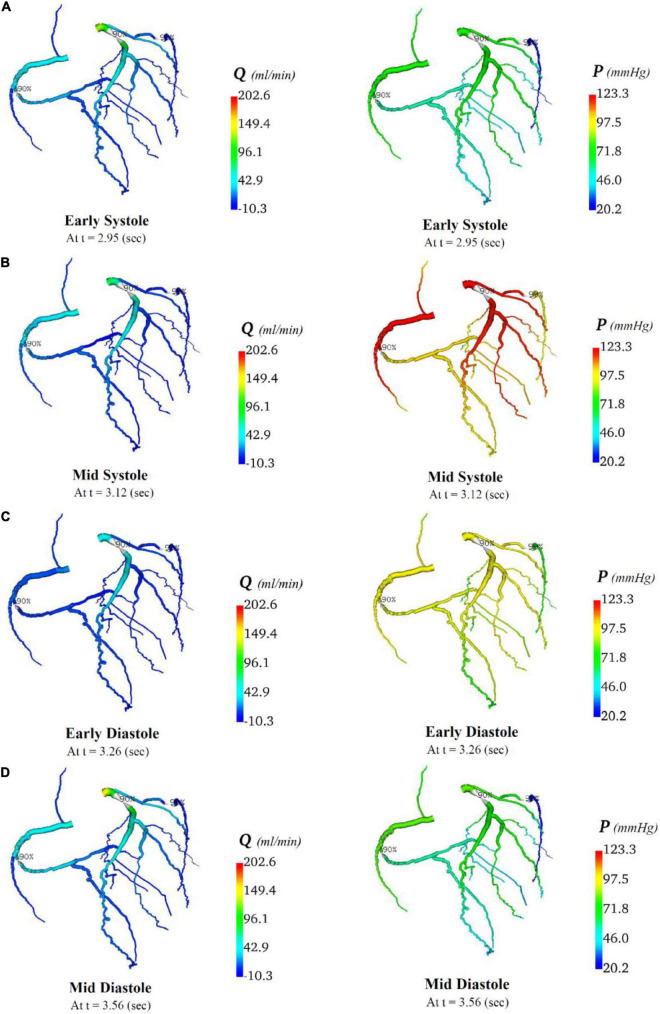
Flows and pressures in stenotic coronary artery circulation. The flows (Q) in ml/min and pressures (P) in mmHg are displayed for four time points in the cardiac cycle: **(A)** early systole, **(B)** mid systole, **(C)** early diastole, and **(D)** mid diastole as indicated in Figure 7C.

**FIGURE 10:**
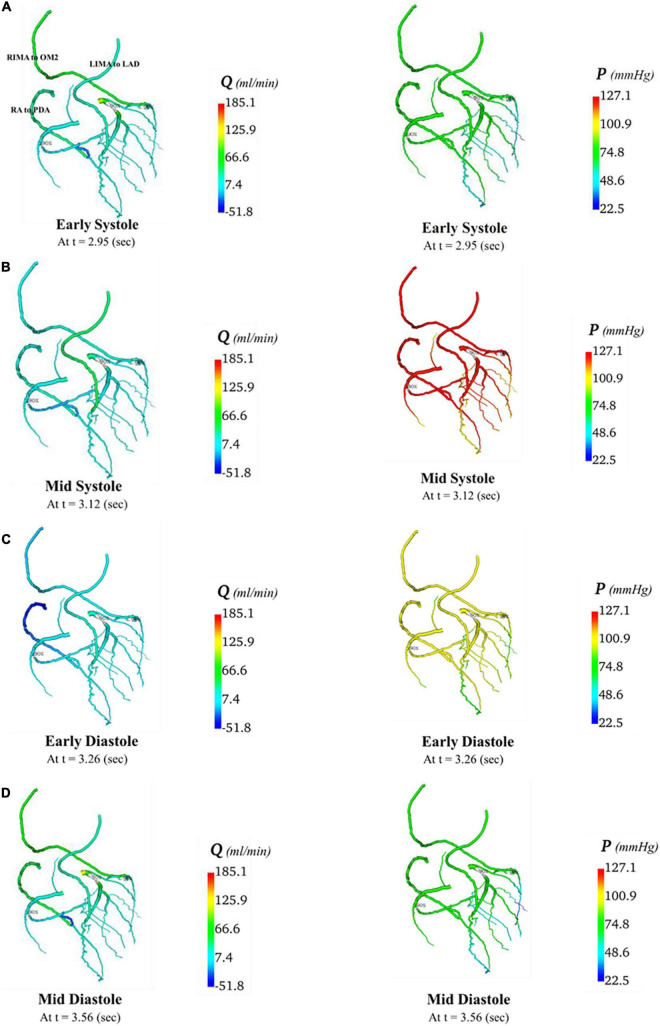
Flows and pressures in grafted circulations with separate graft configuration. The flows (Q) in ml/min and pressures (P) in mmHg are displayed for four time points in the cardiac cycle: **(A)** early systole, **(B)** mid systole, **(C)** early diastole, and **(D)** mid diastole as indicated in Figure 7C: with this virtual graft configuration with an *in situ* LIMA to LAD, *in situ* RIMA *via* transverse sinus to OM2 and RA off aorta to PDA.

**FIGURE 11:**
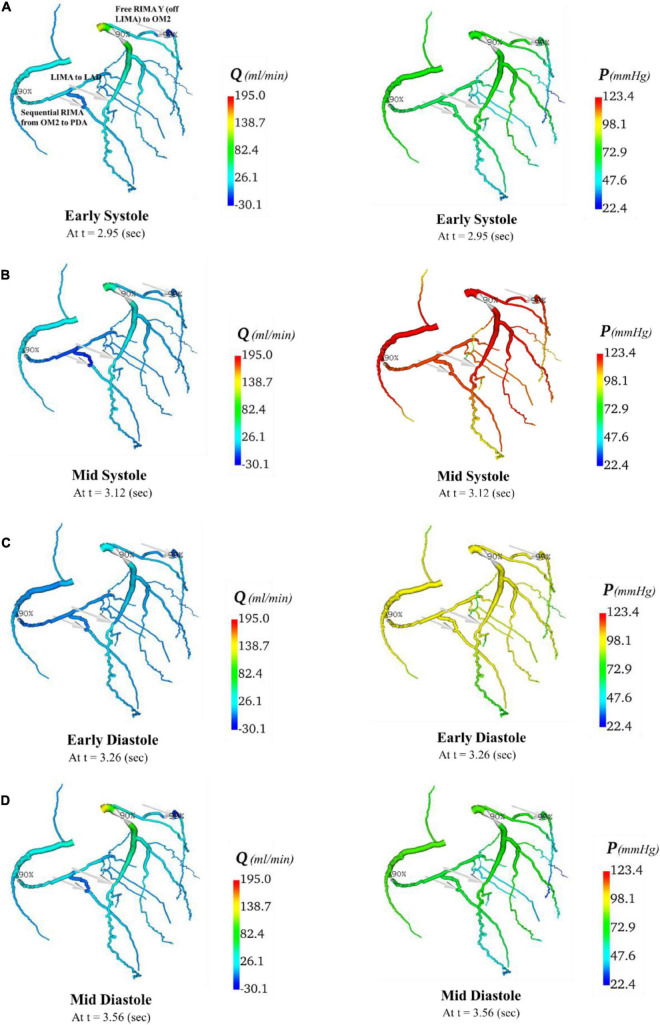
Flows and pressures in grafted circulations with composite graft configuration. The flows (Q) in ml/min and pressures (P) in mmHg are displayed for four time points in the cardiac cycle: **(A)** early systole, **(B)** mid systole, **(C)** early diastole, and **(D)** mid diastole as indicated in Figure 7C: with this virtual graft configuration with *in situ* LIMA to LAD, composite free RIMA Y (off LIMA) to OM2 with RIMA sequential to PDA. The site of the graft anastomosis to the coronary circulation is represented by the grey arrows.

In total, four indicative reference time points (Figure 7C) were used to report the calculated flows and pressures in the cardiac cycle. The flows and pressures obtained according to time throughout the cardiac cycle were interpolated back onto the segmented geometries for visualisation of each patient’s theoretical non-diseased coronary circulation (Figure 8), stenotic circulation (Figure 9), and two virtual grafted circulations. For the separate grafting configuration, the known coordinates of the grafts allowed mapping (Figure 10). For the composite grafting configuration where the coordinates of the grafts were not available, the sites of the grafts were represented by arrows (Figure 11).

### 3.3 Graft performance

**FIGURE 12:**
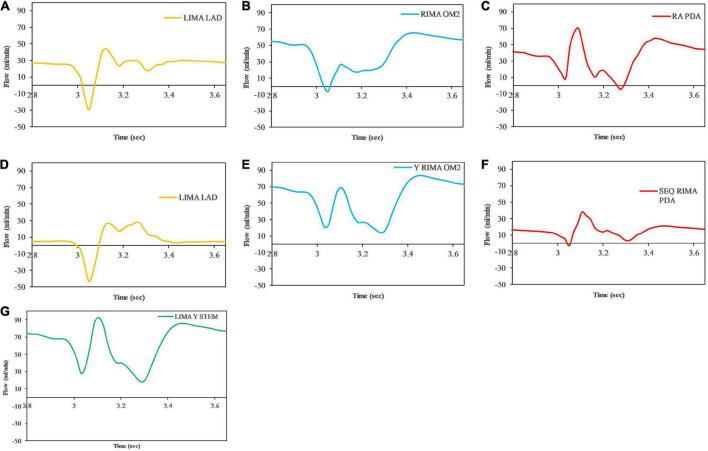
Graft flows between separate and composite configurations as measured at the end of the graft at the anastomosis to the native coronary artery target. For separate grafting arrangement **(A)** LIMA to LAD **(B)** RIMA to OM2 **(C)** RA to PDA and composite grafting arrangement **(D)** LIMA to LAD **(E)** RIMA to OM2 **(F)** RIMA to PDA **(G)** LIMA Y composite common stem. Note that graft flows are presented at the end of the graft (graft outlet). The graft performance indices calculated from these flow waveforms are shown in Table 3.

The graft flow waveforms for one cardiac cycle as measured at the end of the graft are shown for the separate grafting configuration for the *in situ* LIMA to LAD (Figure 12A), *in situ* RIMA to OM2 (Figure 12B), RA off aorta to PDA (Figure 12C) as well as the composite grafting configuration for the composite limb of the LIMA to LAD (Figure 12D), composite Y-graft of the RIMA off the LIMA to the OM2 (Figure 12E), the sequential RIMA graft to the PDA (Figure 12F), and the common stem of the LIMA prior to the Y-graft of the RIMA (Figure 12G). The graft performance parameters that were calculated from these flow waveforms to compare the separate and composite grafting arrangements revealed that the LIMA graft in the composite graft was unsatisfactory with a mean graft flow of 6.31 ml/min at a pulsatility index of 11.33, with backwards flow 20.66% and diastolic filling percentage of 56.04%. In comparison, the separate grafting arrangement had ideal MGF (24.47 ml/min) and PI (2.98) but due to the iFR being 0.93, there was some reverse flows at 3.76%, but the DF was 70.19%. Consequently, the patency of the LIMA to LAD graft would be expected to be better with the separate grafting configuration. Regional perfusion restored by both grafting arrangements was higher than the stenotic circulation; however, separate grafts had more perfusion (Table 3).

## 4 Discussion

### 4.1 Complexity of computational modelling

Although there are a variety of automated, semi-automated, and manual methods for vessel segmentation (89, 90), a manual method was chosen for this model based on the requirement of accuracy and reliability. The major drawback of the manual method was the increased time taken for segmentation which took approximately 6 h. Although automated methods of centreline extraction are significantly more time-efficient, they suffer from image artefact and are prone to errors where the distal vessels are less than 1.5 mm as well as in areas of vessel stenoses (91).

Previous research studies have employed a variety of computational models to investigate coronary artery bypass grafting strategies. These have varied in complexity from simpler 0D lumped parameter models (92, 93) to more sophisticated multi-scale 3D-0D models (81, 94). A significant limitation of models employing only a lumped parameter approach is the oversimplification in representing wave propagation along the larger vessels (84); however, their advantage over 3D models is their relative computational efficiency and ease of implementation. Many of the studies that have utilised 3D models of CABG configurations have used open-source software such as *ANSYS/FLUENT* (94–96) or *OpenFOAM* (97) for implementation. In addition to calculating mean graft flows, these 3D models have also calculated haemodynamic stresses in the coronary circulation such as wall shear stress (WSS) and oscillatory shear index (OSI) which influence long-term graft patency (98). Because of these focii, these research studies have tended to study saphenous vein grafts, sequential grafts, and grafting angles as the outcomes of interest for long-term patency (94, 97, 99). However, in practical terms, grafting angle is not particularly easy for a cardiac surgeon to control whereas WSS and OSI are markers for long-term patency that cannot be easily measured in real time by a surgeon.

In this research, a novel multi-scale 1D-0D lumped parameter patient-specific computational model was developed using a new standalone *Python* package called “COMCAB” created for this purpose. This model was anatomically and physiologically accurate in its ability to predict flow for two different CABG configurations. The model focussed on returning parameters such as MGF, PI, BF, and DF, which most cardiac surgeons are familiar with as they can be measured intraoperatively using the TTFM technology which has been advocated for routine use (100). Low MGF and high PI can indicate competitive flows or poor native coronary run-off affecting the short-term patency of the graft as confirmed on clinical angiographic follow-up (10, 18). For this reason, vessel flows are still the key haemodynamic indicator for the immediate benefit of a procedure, before a whole host of other modulating factors come into play in the longer-term which include haemodynamic stresses such as WSS or OSI that may lead to endothelial dysfunction (101).

Other 1D-0D models of the coronary circulation that have been reported in the literature have been limited to either non-diseased or stenotic coronary circulations (57, 102, 103). Therefore, the treatment of grafting junctions using a 1D CFD approach was a novel feature in this work. The computational model in this study utilised the aortic root beyond the aortic valve as the single inlet for the whole network. This enabled the proximal and distal connections of all bypass grafts to be modelled by including the important systemic aortic branches. Other authors have selectively modelled the distal connections of bypass grafts (104, 105) or used separate isolated inlets for the left coronary artery, right coronary artery, and aorto-coronary bypass grafts (106). Although the focus of the current 1D-0D modelling was the calculation of graft performance indices for use by surgeons, the advantage of the 1D approach is that the model can be developed for future applications such as the study of wave intensity analysis of grafts.

In the computational modelling used for this study, the 0D lumped parameter terminal outlets in the coronary circulation that were connected to the 1D vessels were a 3WK RCR with personalised boundary conditions dictated by the patient-specific coronary geometry. Other studies in the literature have employed more detailed 5-element RCRCR models (35, 55) or even multi-compartmental RCRCR models (57). However, the use of a 3WK RCR model was found to be sufficient for capturing the essential pressure and flow waveforms throughout the coronary circulation. Furthermore, this approach avoided the computationally intensive cost of using a 5-element RCRCR model for assigning resistance and capacitance parameters (35) or the cumbersome parameter fitting involved in trial-and-error (55). The systemic aortic branch outlets have been represented in most studies with a 3WK RCR; however, they have differed in their assignment of the proximal and distal resistances using different set ratios such as *R*_1_ being 9% of the total lumped resistance (55) or 5.6% of the total lumped resistance (64). This set ratio approach does not consider the premature truncation of vessels and thus setting the proximal resistance at the characteristic impedance avoided unnecessary reflections, as described by others (60, 61).

In this research, iFR was applied as the measure for functional significance of stenoses as it obviated the need to run each simulation network at hyperaemia to calculate FFR. Hyperaemic simulations would require prescription of a new inlet pressure boundary condition at the root vessel of the network, as the aortic root pressure is significantly lower in such conditions (103). Furthermore, additional parameterisation for each terminal RCR would be required to account for the decreased resistance at hyperaemia. Previous computational models have used a coronary flow reserve value of 2.6 (107); however, this neglects the patient-specific nature of coronary flow reserve. iFR is gaining favour in clinical practice as an alternative to FFR as it can avoid the risk of adverse reactions to hyperaemic drugs (108).

### 4.2 Separate and composite grafting configurations

The separate grafting configuration had satisfactory flows in the LIMA to LAD graft with MGF 24.47 ml/min, PI 2.98, and DF 70.19%, but the backwards flow was slightly high at 3.76% due to some reverse flow on account of an iFR 0.93 for the LAD stenosis. The composite configuration led to a highly unsatisfactory LIMA to LAD graft with MGF 6.31 ml/min, PI 11.33, BF 20.66%, and poor DF of 56.04% due to a steal of blood flow down the other limb of the composite Y-graft which was accentuated by the native competitive flow in the LAD due to an iFR 0.93 compared to other limbs of the composite graft which supplied coronary vessels with tighter stenoses (iFR 0.29 and iFR 0.73). If a surgeon was to measure the TTFM parameters at the common stem of the LIMA proximal to the take-off of the Y-graft, then the MGF and PI would appear to be satisfactory at 63.86 ml/min and 1.17, respectively. The total myocardial perfusion restored using separate grafts with 3 inflows (238.63 ml/min) was superior to the composite grafting strategy with 2 inflows (227.26 ml/min) (Table 3). However, both grafting configurations exceeded the myocardial perfusion of the stenotic circulation (Table 3). Therefore, without any measurement of intraoperative TTFM, a cardiac surgeon may be led to erroneously believe that both configurations are satisfactory. This highlights the importance of incorporating TTFM parameters into graft assessment. Therefore, a satisficing solution must not only provide adequate blood flows at rest but at favourable mean graft flows, PI and BF to maintain short-term graft patency.

### 4.3 Limitations and future directions

One limitation of the described methodology was the time required to create the patient-specific predictive haemodynamic models. Although the solutions themselves took up to 75 min, the manual segmentation process took up to 6 h. This would limit the practical application of this approach to patients undergoing elective surgery where at least 6 to 12 h would be needed to construct the models and run the solutions. A future aim would thus be more automated, accurate methods of vessel segmentation, and CFD to gain faster solutions. Recently, a deep-learning predictive 3D CABG model which can provide solutions within 1 s has been described (109). However, their deep-learning model did not incorporate the patient-specific nature of the coronary geometry nor assign personalised boundary conditions. Given these limitations, it is questionable whether the computational efficiency in their model justifies the loss of important information.

Another limitation pertained to the geometric parameterisation of the models. Although the geometry of the coronary circulations was patient-specific and personalised, the systemic aortic branch geometry was idealised (28, 29), given the lack of CT scan information regarding the dimensions of these branches. In future studies, this could be overcome by extending the CTCA to include a CTA of the head, neck, thorax, and abdomen. Furthermore, the effects of curvature of vessels including the cross-sectional shape of grafts being elliptical rather than circular near an anastomosis (106) were oversimplified in the 1D-0D model. However, to overcome these limitations would require a 3D model, and these assumptions were not thought to significantly alter the mean flows obtained through the use of the current model, in the context within which they were applied in this study.

Although the 1D-0D lumped parameter computational models used in this research were able to generally capture the key features of the coronary haemodynamics, certain assumptions were required. There was an assumption of Newtonian flow, which some studies have determined to be valid (110) while others have found it to result in important differences (111, 112). The terminal lumped parameter models oversimplified the propagation of waveforms in the microcirculation, but the effects of this on the velocity profile were treated by adjusting the parameters for the Coriolis coefficient. The outlet boundary conditions for the coronary circulations in the computational model were patient-specific; however, the inlet pressure boundary condition, cardiac output, and total arterial compliance were the same for all patients. With more invasive clinical patient data available, these idealised generic parameters could also be made patient-specific.

The verification and validation of the computational model data was established by comparing the predictive haemodynamic results with other 3D computational models in the literature and where available *in vivo* data from other studies in the literature. The assumption of no head loss accentuated the right coronary systolic peak waveform; however, this was mitigated at the point where the vessel branched into the posterior descending artery. However, since surgeons graft more downstream near the distal RCA and PDA, this overestimation did not affect the graft flows near the anastomosis. However, it is acknowledged that a major limitation is the lack of clinical validation using the same patients from whom haemodynamic predictions were made using the computational modelling, rather than comparing against existing clinical data. Therefore, “COMCAB” is currently proposed as a potential tool for use by the surgeons that requires further validation. Such validation is planned in either a large animal model or humans and would require pre-operative iFR calculation of all lesions, measurements of intraoperative TTFM parameters once a grafting configuration has been performed followed by a post-operative CT scan and a myocardial perfusion scan once the subject has recovered adequately from surgery. Post-operative iFR measurements of the grafts and coronary arteries could also be performed. Alternatively, flow information could also be provided by performing pre-operative and post-operative cardiac MRI scans.

Finally, the computational models developed and applied in this study were deterministic in nature, and all haemodynamic calculations were made at basal resting conditions. Thus, the autoregulatory mechanisms in coronary flows were overlooked, as well as the effects of exercise and coronary flow reserve. Some studies have shown that in composite grafts, graft flows and myocardial perfusion at rest may be adequate but at hyperaemia insufficient (113). Other studies have maintained that composite grafts can sustain adequate flows at hyperaemia (114, 115) and this contention requires further investigation in future studies. Furthermore, the variations in flows attributable to cardiac anaesthesia and responses to surgical stimulation including use of cardiopulmonary bypass were not considered (116). Clinical studies have also demonstrated that over time the initial dimensions of the IMA grafts increase, particularly, the common stem in a composite Y-graft becomes larger (117). To overcome such limitations, future stochastic models would need to be investigated to account for variations in flows and graft size diameters under a variety of conditions.

The immediate future directions are to improve the deterministic model including further refinements for efficiency. Thereafter, the coronary artery circulations of more patients can be simulated and a wider variety of grafting configurations can be investigated, in addition to the two configurations presented in this work, to further validate the computational methodology described above. Further clinical validation could be undertaken in either a large animal model or human study. A study could also be conducted to determine the impact of the models on virtual surgical planning by coronary surgeons. A significant advantage of the computational methodology used in this study is the ability to extend to other patient-specific clinical scenarios including personalised vessel geometry, personalised input aortic root pressure waveforms, personalised myocardial resistance and capacitance derived from CTCA volumetric distribution of vessels, personalised graft dimensions, and personalised viscosity parameters that would not be cumbersome. For example, the effects of poorer left ventricular function can be investigated by adjusting cardiac output parameters and the effects of microcirculatory dysfunction evaluated by increasing terminal resistances. With the quest for less invasive diagnostic procedures to detect coronary artery disease such as CTCA (118, 119), an efficient predictive CABG computational model has significant potential to aid surgeons in making better decisions for patients by avoiding unsatisfactory grafting configurations with poor patency.

## Data availability statement

The original contributions presented in this study are included in the article/Supplementary material, further inquiries can be directed to the corresponding author.

## Ethics statement

The studies involving human participants were reviewed and approved by Auckland District Health Board Research Review Committee (ADHB-RRC) A+6900, 23rd September 2015. The patients/participants provided their written informed consent to participate in this study.

## Author contributions

KC and NS conceptualised the computational modelling. AP improved the code efficiency and visualisation of the computational models. KC, AP, and NS edited the manuscript for intellectual content. All authors gave approval for the final version of the manuscript to be published.

## Supplementary material

Supplementary File 1 An excel file containing 4 worksheets with vessel segment data and relations for non-diseased, stenotic, separate grafting, and composite grafting networks.
